# *TOP2A* and *CENPF* are synergistic master regulators activated in cervical cancer

**DOI:** 10.1186/s12920-020-00800-2

**Published:** 2020-10-06

**Authors:** Beiwei Yu, Long Chen, Weina Zhang, Yue Li, Yibiao Zhang, Yuan Gao, Xianlin Teng, Libo Zou, Qian Wang, Hongtao Jia, Xiangtao Liu, Hui Zheng, Ping Hou, Hongyan Yu, Ying Sun, Zhiqin Zhang, Ping Zhang, Liqin Zhang

**Affiliations:** 1 Department of Laboratory, Hangzhou Jianggan District People’s Hospital, Hangzhou, Zhejiang China; 2 Department of Gynecology, Xiao shan Hospital, Hangzhou, Zhejiang China; 3 Department of Gynecology, Qingdao Municipal Hospital, Qingdao, Shandong China; 4 Department of Laboratory, Jinhua People’s Hospital, Jinhua, Zhejiang China; 5 Department of Laboratory, Zhejiang Jinhua Guangfu Hospital, Jinhua, Zhejiang China; 6 Medicine Reproductive Centre, Jinhua People’s Hospital, Jinhua, Zhejiang China; 7 Tianjia Genomes Tech CO., LTD., Hefei, Anhui China; 8 Department of Functional Discipline, School of medicine Jinhua, Jinhua, Zhejiang China

**Keywords:** Cervical cancer, Master regulator, Transcriptome, Differential expression, Differential activity

## Abstract

**Background:**

Identification of master regulators (MRs) using transcriptome data in cervical cancer (CC) could help us to develop biomarkers and find novel drug targets to fight this disease.

**Methods:**

We performed differential expression (DE) analyses of public microarray and RNA-seq transcriptome data of CC and normal cervical tissues (N). Virtual Inference of Protein activity by Enriched Regulon analysis (VIPER) was used to convert the DE outcomes to differential activity (DA) signature for MRs. Synergy analysis was conducted to study synergistic effect of MR-pairs. TCGA and microarray data were used to test the association of expression of a MR and a clinical feature or a molecular feature (e.g. somatic mutations). Various bioinformatic tools/websites (DAVID, GEPIA2, Oncomine, cBioPortal) were used to analyze the expression of the top MRs and their regulons.

**Results:**

Ten DE and 10 DA signatures were generated for CC. Two MRs, DNA topoisomerase II alpha (*TOP2A*) and centromere protein F (*CENPF*) were found to be up-regulated, activated and synergistic in CC compared to N across the 10 datasets. The two MRs activate a common set of genes (regulons) with functions in cell cycle, chromosome, DNA damage etc. Higher expression of *CENPF* was associated with metastasis. High expression of both MRs is associated with somatic mutation of a set of genes including tumor suppressors (*TP53*, *MSH2*, *RB1*) and genes involved in cancer pathways, cell cycle, DNA damage and repair. The magnitude of up-regulation and the absolute expression level of both MRs in CC are significantly higher compared to many other cancer types.

**Conclusion:**

*TOP2A* and *CENPF* are a synergistic pair of MRs that are overexpressed and activated in CC. Their high expression is correlated with some prognosis features (e.g. metastasis) and molecular features (e.g. somatic mutations) and distinctly high in CC vs. many other cancer types. They may be good biomarkers and anticancer drug targets for CC.

## Background

Cervical cancer (CC) is the fourth most common female malignancy worldwide. More than half a million women are diagnosed with cervical cancer and the disease results in over 300,000 deaths worldwide each year [1, 2]. Extended understanding of the gene expression programs, especially the master regulators (MRs) in CC, will help us to fight this devastating disease.

Since early 2000s, microarrays have been used to profile transcriptome of cervical cancer tissues. Further, the application of RNA-sequencing (RNAseq) in recent years has rapidly generated large amount of data on the gene expression landscape of this disease. Differential expression (DE) analysis is the most common and straightforward analysis for a gene expression dataset. Through DE analysis, a set of highly up-regulated and down-regulated genes can be identified by comparing two groups of samples (e.g. cancer vs. normal). However, gene expression is highly dynamic, and the expression quantification may depend on the techniques (e.g. different platforms of microarray, or RNAseq), making the cross-dataset comparison difficult. Moreover, the top regulated gene may not represent the key genes or the MRs under a biological system. The activities of MRs cannot be directly measured by microarray or RNA-seq because these techniques only measure RNA expression level and do not consider protein level and protein activity changes by post-translational modifications. Fortunately, MR activity can be inferred by its regulons (genes downstream and regulated by of the MR) through the “Virtual Inference of Protein activity by Enriched Regulon analysis” (VIPER) [3], which takes the DE data and an context-specific gene regulatory network (interactome) as inputs.

The presence of MRs in various cancers has been well-documented, whose coordinated activity within tightly regulated modules (tumor checkpoints) are strictly necessary for tumor state initiation and maintenance [3]. To identify the top MRs in CC, we initially collected 10 transcriptome datasets from Gene Expression Omnibus (GEO) [4] with CC and normal cervical tissue (N) samples. We performed gene expression analysis using CC compared to N and generated 10 DE signatures. Then VIPER was conducted to convert the 10 DE signatures to 10 differential activity (DA) signatures for MRs. We further analyzed the DA-DE consistency, the synergy of MR pairs and identified *TOP2A*-*CENPF* as a synergistic MR pair in CC. Both MRs showed increased activity and expression. We further studied the common regulons of the two MRs, the links between the expression of the two MRs and clinical or molecular features of CC. Finally, we compared the expression of them in CC vs. other cancer types.

## Methods

### GEO data collection

Data were collected through searching Gene Expression Omnibus (GEO) [4] for microarray or RNA-seq data for gene expression profiling using key word “cervical cancer”. All datasets were manually inspected to select datasets with both cervical cancer (CC) tissue samples and normal (N) cervical tissue samples. To eliminate the potential noise from small number of samples, a minimum of 5 samples per group was required for each dataset. A summary of all data used can be found in Table 1.

**Table 1:** Datasets used in this study

Data Name	GEO ID	#Tumor	#Normal	Platform	Dataset type	Ref^a^
Biewenga_2008	GSE7410	19	5	Agilent Oligo G4112A	discovery	[5]
Boon_2015	GSE63514	28	24	Affymetrix U133 Plus 2.0	discovery	[6]
Guardado_2012	GSE29570	45	17	Affymetrix Gene 1.0 ST	discovery	[7]
Medina_2014	GSE52903	55	17	Affymetrix Gene 1.0 ST	discovery	[8]
Zhai_2007	GSE7803	21	10	Affymetrix U133A	discovery	[9]
Li_2018	GSE107472	5	5	RNA-seq	validation	[10]
Pappa_2015	GSE63678	5	5	Affymetrix HG133_A_2.0	validation	[11]
Pyeon_2007	GSE6791	20	8	Affymetrix U133 Plus 2.0	validation	[12]
Scotto_2008	GSE9750	33	24	Affymetrix U133A	validation	[13]
Sun_2014	GSE55940	5	5	Glue Grant Transcriptome 3.0	validation	[14]

^a^*Ref*. references

### Gene expression analysis of microarray data from GEO database

Gene expression of microarray data were computed using the GEO2R tool (https://www.ncbi.nlm.nih.gov/geo/geo2r/). The CC and N samples within the same dataset were compared. Normalized expression values of genes or probes were visualized within GEO2R tool as a boxplot. Obvious outliers (samples with distribution of normalized values very different from the rest of samples) were excluded from the dataset for further analysis. Statistical test results for all probes were downloaded. Moderated *t*-statistic were used as the DE measurement for the dataset. If one gene has multiple probes, only the one with highest absolute *t* value was used. All probes without assigned gene ID were discarded. For dataset Biewenga_2008 [5], the expression values for an individual gene/probe for all samples were extracted using the “Profile graph” function in GEO2R tool. The expression differences between different sample types (ie. N, Normal; C, tumour without lymph node metastasis; M, tumour with lymph node metastasis) were analyzed using Wilcoxon Rank-Sum test.

### Gene expression analysis of RNA-seq data from GEO database

Fastq files were downloaded from Sequence Read Archive (SRA, https://www.ncbi.nlm.nih.gov/sra/, [15]) database using SRA Toolkit (https://ncbi.github.io/sra-tools/). Reads were mapped to human reference genome (hg19) using STAR (2.6.0a) [16]. Reads with mapping quality score (MAPQ) < 10 or those with > 5 mismatches in 100 bp aligned region were discarded. Reads mapped to coding sequence (CDS) region of Refseq-defined genes were used to quantify gene expression. For non-protein-coding transcripts, reads mapped to all exons were used to quantify gene expression. For genes with multiple transcripts, only the transcript with the largest number of reads (sum for all samples) was used. DESeq2 [17] was used for differential expression (DE) analysis comparing CC vs. N samples. The DE measurement for RNA-seq is -S × log10 (adjusted *P*-value), in which S is the sign indicating the direction of regulation (+ 1 for upregulation and − 1 for downregulation).

### Master regulator (MR) analysis using VIPER

Virtual inference of protein activity by enriched regulon analysis (VIPER) [3] was conducted to infer the master regulator differential activities (DA) from the DE data. The “msviper” function in R package “viper” was used as the program to run VIPER analysis. VIPER takes a DE signature and a regulatory network as inputs. Context-specific regulatory networks “aracne.networks” [18] were downloaded from Bioconductor. Human cervical squamous carcinoma context-specific ARACNe interactome “reguloncesc” was used as the “network” in this study. Parameter “pleiotropy” was set to TRUE to correct for the effects of target overlap between different MRs. VIPER outputs *P*-values and normalized enrichment scores (NES) which represents the DA signature of MRs.

### MR categories

These 5838 MRs in interactome “reguloncesc” were categorized into three groups based on their function annotation in gene ontology (GO) database (http://geneontology.org/): (1) 1588 transcriptional factors (TFs): genes annotated in the GO Molecular Function database GO:0003700 (DNA binding transcription factor activity), or GO:0003677 (DNA binding) and GO:0030528 (Transcription regulator activity), or GO:0003677 and GO:0045449 (Regulation of transcription); (2) 280 transcriptional co-factors (TFcoFac): genes not in TF and annotated as GO:0003712 (transcription cofactor activity), GO:0030528 or GO:0045449; (3) 3883 in signal pathways (SigPathway): genes not in TF or TFcoFac and annotated in the GO Biological Process database as GO:0007165 (signal transduction) or in the GO Cellular Component database as GO:0005622 (intracellular) or GO:0005886 (plasma membrane); 87 MRs were not annotated in these three categories.

### Selecting common top MRs in CC

The 10 GEO datasets (Table 1) were randomly assigned to two groups: 5 discovery datasets and 5 validation datasets. To select top common MRs in CC, the top N (*N* = 50, 100, 200, 300) most activated MRs and top N suppressed MRs in each one of five discovery datasets were selected. The common activated and suppressed MRs were the common ones in the top N MRs from all the five discovery datasets. Whether these common top MRs were also significant in the validation datasets were examined by comparing the DA values of the common MRs vs. all the other MRs using Wilcoxon Rank-Sum test. We also require that these top MRs have significant and consistent DE change in the discovery datasets. With another requirement that DE > 3 or DE < -3 for when the DA is positive or negative respectively, the resulting MRs are called DA-DE consistent MRs. In this study, the results for *N* = 100 were presented in the main text and results for all Ns (*N* = 50, 100, 200, 300) are provided in the supplementary Table 1.

### Synergy analysis of MR pairs

Synergy of two MRs was to test whether their common regulons have a more extreme DE signature than the rest of regulons. Among the DA-DE consistent MRs, all pairs of MRs were considered for synergy analysis. For a given pair of MRs (denoted here as MR1 and MR2), their regulons were extracted from the interactome “reguloncesc” in regulatory networks “aracne.networks” [18]. Then the common regulons for both MRs were selected. Only when MR1 and MR2 have > 10 and > 10% common regulons among the regulon number in both MR1 and MR2, the synergy analysis was conducted. All regulons belong to MR1 or MR2 were separated to three groups: common, MR1-unique and MR2-unique. For each DE dataset, two statistical tests were performed: common vs. MR1-unique for MR1 regulons and common vs. MR2-unique for MR2 regulons. The Expression Contribution to Activity (ECA) values of all regulons were calculated for each MR and each DE dataset. ECA = DE x MOR, in which DE is the differential expression value and MOR is the mode of regulation value of a MR-regulon pair from the interactome “reguloncesc”. MOR indicates the sign of the association between a MR (regulator) and a regulon (target gene) and ranges between − 1 and + 1, with positive and negative value indicating positive and negative regulation respectively. The ECA distribution of common regulons were compared with MR1-unique or MR2-unique regulons, using Wilcoxon Rank-Sum test (one tailed), in which the alternative hypothesis of the test is “greater” and “less” when the sign of the DA of the MR in the DE dataset is positive and negative respectively. A synergistic MR pair will show significant higher ECA values for common regulons than other regulons if the MR is activated (positive DA value) and vice versa.

### Network visualization of MRs and regulons

The MR and regulon network were visualized using Cytoscape 3.7.1 [19]. A node is either a MR or a regulon. An edge is a MR-regulon relationship. Node color represents the average DE scores of a gene (a MR or a regulon) in the 10 datasets with red and blue indicating up-regulation and down-regulation respectively. Edge color represent the MOR of a MR and a regulon relationship with red and blue indicating positive and negative regulation respectively.

### DAVID analysis

The function annotation analysis for a given gene list was conducted using the Database for Annotation, Visualization and Integrated Discovery (DAVID) v6.8 (https://david.ncifcrf.gov/) [20]. The gene symbols of a gene list were submitted to the DAVID webserver. “OFFICIAL_GENE_SYMBOL” was chosen to map the genes to the IDs in the DAVID database. The annotations of genes were limited to “*Homo sapiens*” only. The background gene list was also set to “*Homo sapiens*”. The “Functional Annotation Clustering” function of the website was taken. Several gene annotation databases were included: UP_KEYWORDS, UniProt Keywords; UP_SEQ_FEATURE, UniProt sequence feature; GOTERM_BP_DIRECT, Gene ontology biological process; GOTERM_CC_DIRECT, Gene ontology cellular component; KEGG_PATHWAY, KEGG pathway. The results were downloaded and certain generic annotation terms with total gene number (“Pop Hits”) > 1000 were removed.

### TCGA data

The gene expression data for individual genes for CC samples in The Cancer Genome Atlas (TCGA) [21] was retrieved from TSVdb [22]. The RSEM (RNA-Seq by Expectation Maximization) values were used. The overall survival (OS) data was also retrieved from TSVdb. Other clinical and molecular features (e.g. Somatic mutation data) data were retrieved from the supplementary Table 1 and 2 of Cancer Genome Atlas Research Network et al., 2017 [23]. The loss-of-function (LOF) mutations include nonsense, frame shift and splicing mutations. A sample with both a missense mutation and a LOF mutation of a gene is classified as LOF. The description of each clinical or molecular feature can be found in the “features list” tab in the supplementary Table 1 of Cancer Genome Atlas Research Network et al., 2017 [23]. The features were separated to categorical or numerical data. For each categorical feature, only the sample groups with > 5 samples were analyzed. The expression values of a MR for different groups of a categorical feature were compared using Wilcoxon Rank-Sum test. All gene somatic mutation data were treated as categorical data with three groups: no mutation (None), missense mutation and LOF mutation. Rather than setting a minimal sample number threshold to filter the data, we selected all genes with missense or LOF mutations having expression of a MR different than the None group with *P* < 0.1. For each numerical feature, the Spearman correlation was computed to test the significance of the correlation of the expression of a MR and the feature.

For survival analysis, all patients with OS data were divided into two groups based on the 80th percentile value of the expression of a gene. The survival curve was drawn using the R package “survminer” (https://cran.r-project.org/web/packages/survminer/). *P*-value is calculated based on the log-rank test.

### Network visualization of association of MRs and genes with somatic mutations

The MR and somatic mutation gene network were visualized using Cytoscape 3.7.1 [19]. A node is either a MR or a gene with somatic mutations (SMG) in TCGA CC samples. An edge is a MR-SMG relationship. Edge color represents the direction of a MR-SMG relationship with red and blue indicating that samples with a SMG with mutations associated with high and low expression of a MR respectively. The arrowhead shape represents the type of a mutation with an open square and a solid diamond representing a missense mutation and a LOF mutation respectively.

### GEPIA2 analysis

Gene Expression Profiling Interactive Analysis 2 (GEPIA2) is a web server for analyzing the RNA sequencing expression data of 9736 tumors and 8587 normal samples from the TCGA and the GTEx projects (http://gepia2.cancer-pku.cn) [24]. We analyzed the expression of the top MRs in cancer and normal tissues for 33 cancer types using the “Dot plot” function of GEPIA2. The expression of a gene is plotted in log2(TPM + 1) scale, in which TPM is the Transcript Per Million value. Analysis of variance (ANOVA) was used to test the statistical significance of the difference of expression in tumor tissues vs. paired normal tissues.

### Oncomine analysis

Oncomine is a bioinformatics platform aimed at collecting, standardizing and analyzing cancer transcriptome microarray data (http://www.oncomine.org) [25]. We used Oncomine 4.5 to analyze the expression of top MRs (*CENPF* and *TOP2A*) in different cancers types. All datasets were filtered using the gene symbol, “Analysis Type = Cancer vs. Cancer Analysis” and “Cancer Type = cervical cancer”. The “Bittner Multi-cancer” dataset was returned as the top significant dataset. “Bittner Multi-cancer” is from the Expression Project for Oncology (expO) which contains 1911 various tumor samples analyzed on Affymetrix U133 Plus 2.0 microarrays (unpublished, GEO ID, GSE2109). The log2 median-centered intensity was used as gene expression value. The expression difference of a MR in cervical cancer vs. other cancer types was tested using Student’s *t*-test.

### The human protein atlas

The immunohistochemistry of cervical cancer tissue, protein subcellular localization and cell cycle expression pattern information were extracted from the “PATHOLOGY” and “CELL” tab of The Human Protein Atlas (http://www.proteinatlas.org) [26]. The cell cycle dependency expression data for a protein was analyzed using a custom assay by staining of U-2 OS FUCCI cells. The FUCCI cells, Fluorescence Ubiquitination Cell Cycle Indicator, are cells tagged with different fluorescent proteins fused to two different cell cycle regulators Cdt1 (expressed in G1 phase) and Geminin (expressed in S and G2 phases) that allows cell cycle monitoring. When both proteins are present, the overlay of the images appear in yellow marking the G1/S transition.

### OncoPrint visualization using cBioPortal

OncoPrints are compact means of visualizing distinct genomic alterations, including somatic mutations, copy number alterations, and mRNA expression changes across a set of cases. OncoPrint was generated using cBioPortal [27].

### Protein-protein association analysis using STRING

STRING [28] was used to study the protein-protein association of TOP2A, CENPF and their common regulons with average DE score (DE.average) > 5. Only associations experimentally determined, from curated database or from text mining were shown. The kmeans clustering method with the number of clusters = 3 was used for the clustering analysis.

### DepMap analysis

The correlation analysis of gene expression and promoter methylation for TOP2A and CENPF was performed using the Data Explorer tool of DepMap [29].

### Expression signature comparison with the connectivity map (CMap) using CLUE

The Connectivity Map (CMap) [30] is a database developed by the Broad Institute with > 1 million expression signatures of cells responding to chemical, genetic, and disease perturbations. CLUE (https://clue.io/) [30] is a cloud-based software platform for the analysis of perturbational datasets of CMap. QUERY is a tool in CLUE to find positive and negative connections between the gene expression signature of interest and all the signatures in CMap. We used TOP2A, CENPF and their common regulons with average DE score (DE.average) > 5 as input upregulated gene list and used QUERY tool to query the CMap database. The result included connectivity score matrix of 8969 perturbations in 9 cell types. The connectivity score is a standardized measure ranging from − 100 to 100 with a positive score indicating a similarity between a given perturbagen’s signature and that of the query, while a negative score indicating an opposing signature.

### Statistical test

All statistical tests and plotting were performed in R 3.5.2 (https://www.r-project.org/) unless otherwise mentioned.

## Results

### Identification of top MRs in CC

**Fig. 1:**
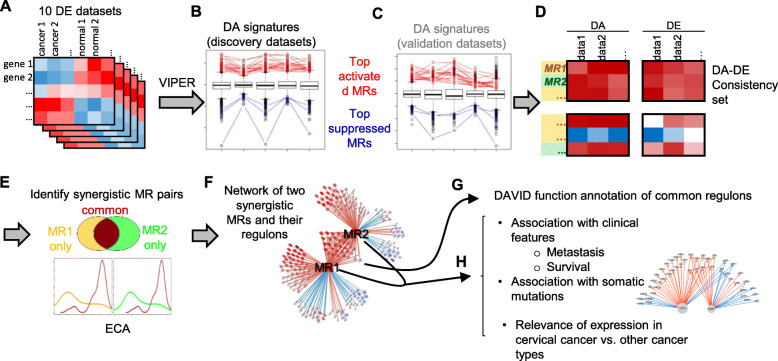
A flowchart of current study strategy to analyze the MRs of CC. **a** 10 gene expression datasets of cervical cancer tissues and normal cervical tissues were selected from GEO database. **b** VIPER was used to convert a DE profile of genes to a DA profile of MRs. Top activated or suppressed MRs were selected from 5 discovery datasets. **c** The top MRs were validated in the other 5 validation datasets. **d** the DE profiles of the top MRs in the 5 discovery datasets were extracted. DA-DE consistent MRs were selected. **e** Synergistic MR pairs were identified by comparing the distribution of ECA for common and unique regulons of two MRs. **f** The top significant synergistic MR pair and their regulons were visualized in a network. **g** The common regulons of the two MRs were functionally annotated using DAVID. **h** The clinical significance the two MRs was further analyzed using additional data such as TCGA

**Fig. 2:**
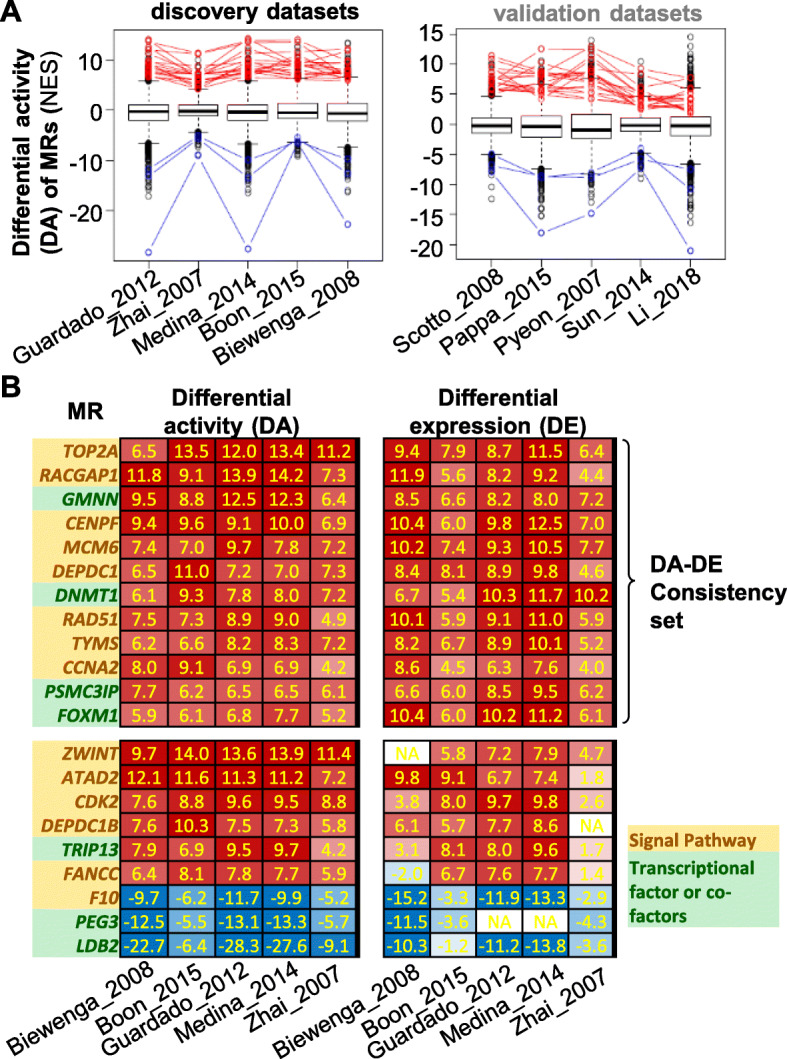
Identification of top MRs in CC. **a** Left, Top 100 activated MRs and 100 suppressed MRs were selected within each of the five discovery datasets. The common 18 activated and 3 suppressed MRs were identified and shown as red and blue in the NES boxplot. Right, the same 18 and 3 MRs were shown in the NES boxplot of other 5 validation datasets. The NES value distribution of the 18 and 3 MRs compared to the rest of MRs were tested using Wilcoxon Rank-Sum test. The *P*-values are 2e− 13, 2e− 13, 2e− 13, 2e− 13, 2e− 13 for the 18 activated MRs for discovery set; 1e− 03, 1e− 03, 1e− 03, 1e− 03, 1e− 03 for the 3 suppressed MRs for the discovery set; 1e− 13, 4e− 13, 1e− 12, 3e− 12, 9e− 11 for the 18 activated MRs for validation set; 2e− 03, 1e− 03, 1e− 03, 2e− 03, 1e− 03 for the 3 suppressed MRs for the validation set. **b** The DA (NES scores) and DE (*t-*scores) matrix of the 21 MRs (18 activated and 3 suppressed) in the 5 discovery datasets. DA-DE Consistency set was selected using |DE| value> 3 and same sign as DA in all five datasets. MRs in signal pathway or transcription factors were highlighted in orange and green background respectively

To identify the top MRs in CC, a collection of 10 datasets were selected from GEO database (Table 1). Each dataset has at least 5 CC samples and at least 5 normal cervical tissues as controls. Alltogether, 236 CC samples and 120 normal tissues were analyzed. The DE analysis was performed in each of the 10 datasets, resulting in 10 DE signatures. Using VIPER [3], we converted each DE signature of all genes to a DA signature of 5838 MRs (Fig. 1a-c, supplementary Table 1). We sought to use a discovery-and-validation approach (Fig. 1b-c) to identify top MRs. The 10 datasets were randomly assigned to a discovery group and a validation group each include 5 datasets (Table 1). Using the top 100 most activated and top 100 most suppressed MRs in each of the 5 discovery DA signatures, we identified 18 common activated and 3 common suppressed MRs (Fig. 2a left). The 18 and 3 MRs were found to be also significantly enriched in the top activated and suppressed MRs, respectively in the 5 validation datasets (Fig. 2a right), indicating that these MRs are robust top MRs in CC. Next, we compared the DA and DE signatures of the 18 and 3 MRs (Fig. 1d) using the 5 discovery datasets and selected 12 MRs which have consistent DA and DE profiles. All the 12 MRs are activated and up-regulated (Fig. 2b). The 12 MRs contain four transcriptional factors or co-factors (*GMNN*, *DNMT1*, *PSMC3IP*, *FOXM1*) and eight involved in signal pathways (*TOP2A*, *RACGAP1*, *CENPF*, *MCM6*, *DEPDC1*, *RAD51*, *TYMS*, *CCNA2*) (Fig. 2 b).

### Identification of synergistic MRs *TOP2A* and *CENPF* in CC

**Fig. 3:**
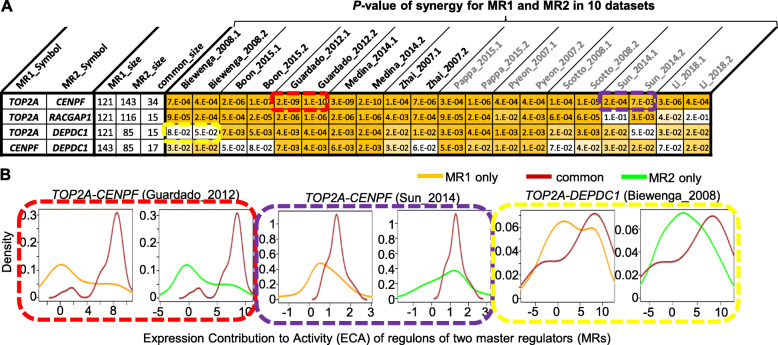
Identification of synergistic MR pairs. **a** Each row shows a pair of MRs (MR1 and MR2), their sizes (number of regulons) and common size (number of common regulons), two Wilcoxon Rank-Sum test *P*-values for each dataset. **b** Six examples of synergistic tests as shown in the color-matched boxes in A. For each MR-pair in a dataset, the ECA values of the common regulons were compared to either MR1-unique regulons or MR2-unique regulons, generating two *P*-values

**Fig. 4:**
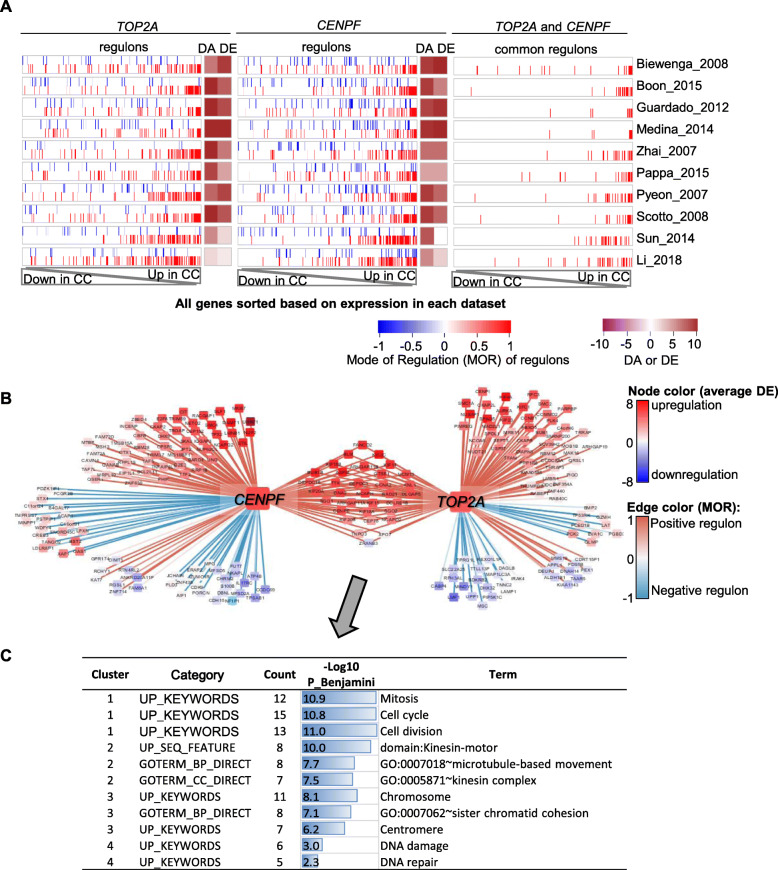
Synergistic effect of *TOP2A* and *CENPF*. **a** Regulon enrichment plot of regulons of *TOP2A* (left), *CENPF* (middle) or common regulons (right). For each dataset, all genes were sorted based on the DE signature from most down-regulated to most up-regulated. The position of regulons in the sorted gene list was shown as a vertical blue (negative regulons) or red (positive regulons) bar. **b** Network view of *TOP2A*, *CENPF* and their regulons. Node color represents the average DE signature in the 10 datasets. Edge color represent the MOR of a MR and a regulon. **c** DAVID function annotation of the common regulons of *TOP2A* and *CENPF*

Next, we sought to identify synergistic MR-pairs in CC, i.e., the two MRs with their common regulons being more extremely regulated than regulons unique to each of them (Fig. 1e-f). From the 12 DA-DE consistent MRs, four pairs have at least an overlap of 10 regulons and 10% of regulons (Fig. 3a). The synergy test was conducted using the ECA values of common regulons vs. MR1- or MR2-unique regulons using Wilcoxon Rank-Sum test for the 10 datasets (Fig. 3a-b). MR pair *TOP2A* and *CENPF* was found to be highly significant in all the 10 datasets (*P*-value range from 7E-3 to 1E-10). The common regulons of them are all positively regulated (MOR> 0) by both MRs and were more up-regulated than the rest of regulons (Fig. 4a). There are all together 34 common regulons of *TOP2A* and *CENPF* (supplementary Table 2, 3). Most of them (32) are upregulated in all the 10 datasets with an average DE > 2 (Fig. 4b, supplementary Table 2). A functional annotation of the common regulons showed that their encoded proteins are significantly associated with (1) mitosis, cell cycle, cell division; (2) kinesin-motor, microtubule-based movement, kinesin complex; (3) chromosome, sister chromatid cohesion, centromere; (4) DNA damage, DNA repair (Fig. 4c, supplementary Table 4).

### Clinical implications of expression of *TOP2A* and *CENPF* in CC

**Fig. 5:**
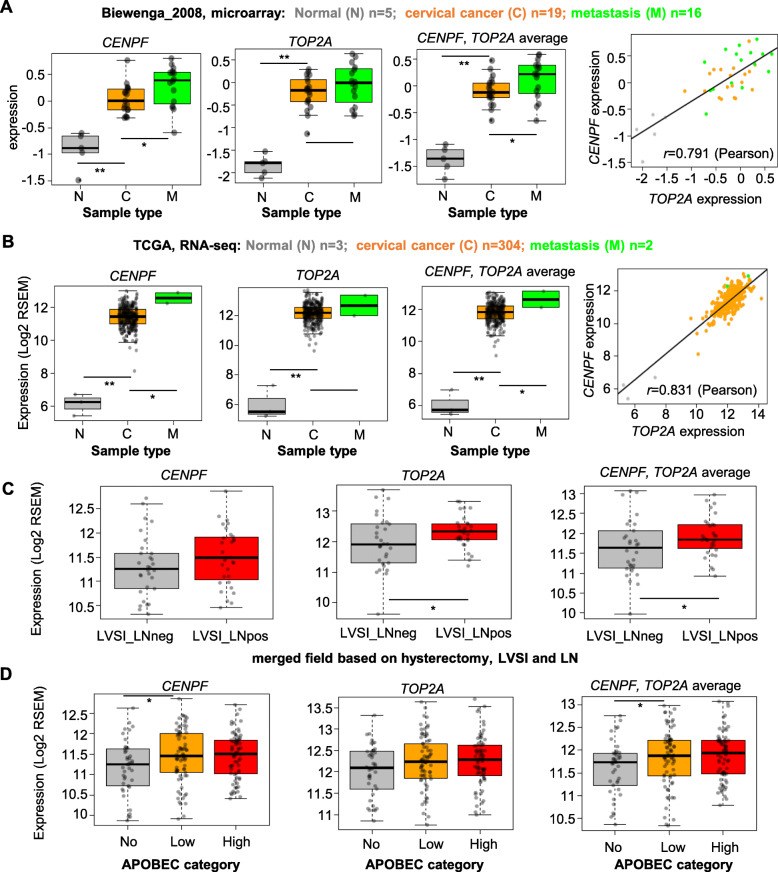
*TOP2A* and *CENPF* expression is correlated with clinical or molecular features. **a** Left three plots, expression of *CENPF*, *TOP2A* and the average of the two for different sample types in Biewenga_2008 dataset. *P*-values were from Wilcoxon Rank-Sum test. *, *P* < 0.05; **, *P* < 0.01. Right, scatter plot of the expression of the two genes. The Pearson correlation coefficient is indicated in the plot. **b** Similar to A, except that TCGA RNAseq gene expression data was used. **c** Similar to B, except that two clinical categories of hysterectomy, LVSI and LN (LVSI_LNneg and LVSI_LNpos) were compared. **d** Similar to B, except that three APOBEC categories were used to group samples

Next, we explored the question whether the expression of *TOP2A* and *CENPF* have any clinical implications. We used Biewenga_2008 (Table 1) microarray data which has 5 normal cervical tissues (N), 19 tumour samples without lymph node metastasis (C) and 16 tumour samples with lymph node metastasis (M) (supplementary Table 5). As expected, both *CENPF* and *TOP2A* expression are significantly (*P* < 0.01) higher in C vs. N. Interestingly, both *CENPF* and the average of the two genes showed higher expression (*P* < 0.05) in M vs. C. (Fig. 5a). The median expression of *TOP2A* is also higher in M vs. C, although the *P*-value is not significant. The expression of *CENPF* and *TOP2A* is highly correlated (Pearson correlation coefficient = 0.79) in all the samples. These observations were confirmed using the TCGA data (RNA-seq), which has 3 N samples, 304 C samples and 2 M samples (Fig. 5b, supplementary Table 6). From a survey of additional 21 clinical or molecular features (including 14 categorical and 7 numerical features, supplementary Figure 1, 2), we found that clinical feature lymphovascular space invasion (LVSI) and lymphnodes (LN) positive is associated with higher (*P* < 0.05) expression of *TOP2A* or the average expression of the two genes in CC tissues (Fig. 5c, supplementary Table 6). The samples with molecular feature APOBEC mutagenesis (Low) has higher expression of *CENPF* or the average of the two genes (*P* < 0.05) than the samples without APOBEC mutations (No) (Fig. 5d, supplementary Table 6). The OS of samples with high expression of *CENPF* is slightly poorer than those with lower expression, although the *P*-value did not reach significance level (*P* = 0.15, supplementary Figure 3). Among all the numerical features, only the sample purity computed by ABSOLUTE (Purity_Absolute) showed positive correlation with the expression of *CENPF* (*P* = 0.03) and *TOP2A* (*P* = 3E-5) (supplementary Figure 2), confirming the finding that these genes are highly up-regulated in CC compared to N tissues. Other features, including race, human papillomavirus (HPV) status or type, clinical stage, EMT score, mutation rate, etc. do not correlate with the expression of the two genes (supplementary Figure 1, 2, supplementary Table 6).

### Somatic mutations associated with expression of *TOP2A* and *CENPF*

**Fig. 6:**
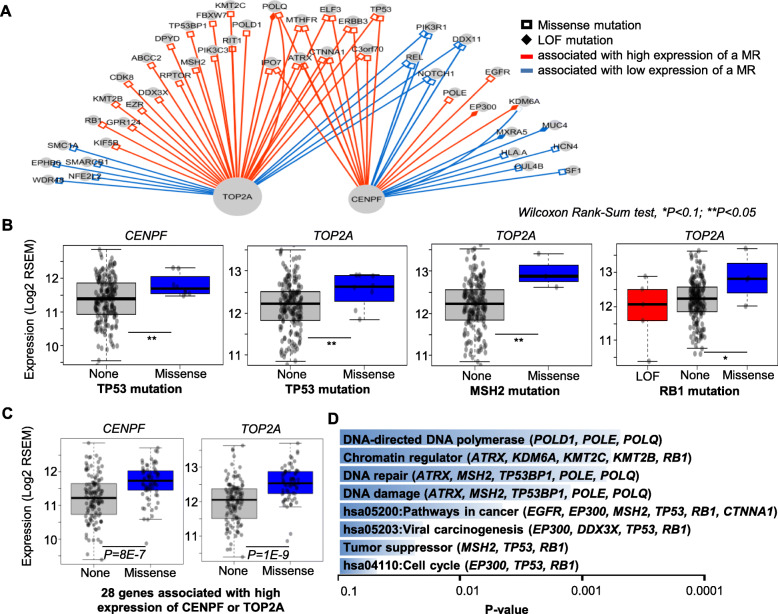
Tumor samples with high or low expression of *CENPF* and *TOP2A* are associated with gene somatic mutations. **a** Network view of *CENPF*, *TOP2A* and genes with somatic mutations in TCGA samples with CC. Wilcoxon Rank-Sum test *P* < 0.1 was used to select associations between a type of mutation and gene expression of a MR. Loss of function (LOF) mutations include nonsense, splice site mutations and frameshift mutations. **b** Examples of mutations in three genes (*TP53*, *MSH2* and *RB1*) associated with expression level of *CENPF* or *TOP2A*. (two tailed Wilcoxon Rank-Sum test, * *P* < 0.1, ** *P* < 0.05) **c** Expression of *CENPF* or *TOP2A* in 67 samples with missense mutations in any of the 28 genes compared to 124 samples with none of these mutations. **d** DAVID function annotation of the genes with mutations associated with high expression of *CENPF* or *TOP2A*

The association of the expression of *CENPF* and the APOBEC mutagenesis (Fig. 5d) prompted us to find genes with somatic mutations (SMGs) associated with the expression of the two MRs. Using the TCGA data, we identified 45 SMGs forming 60 SMG-MR pairs, in which a SMG with either missense or LOF mutations associate with high or low expression of a MR (*TOP2A* or *CENPF*) with *P* < 0.1 (Wilcoxon Rank-Sum test) (Fig. 6a, supplementary Table 7). Majority (55/60) of these associations involve missense mutations. And two third (40/60) of the mutations are correlated with higher expression of the two MRs (supplementary Table 7). For example, missense mutations of *TP53* gene are associated with higher expression of both *CENPF* and *TOP2A* (*P* < 0.05) (Fig. 6b left two). Missense mutations of *MSH2* (*P* < 0.05) or *RB1* (*P* < 0.1) are associated with higher expression of *TOP2A* (Fig. 6b right two). Altogether missense mutations of 28 genes are associated with higher expression of *CENPF* or *TOP2A*, among them 9 are associated with higher expression of both MRs (Fig. 6a). By grouping samples based on a combined effect of all the 28 genes, samples with missense mutations in any one of these genes have significantly higher (*P* = 8E-7 and 1E-9 respectively) expression of both MRs (Fig. 6c). A functional annotation analysis using DAVID showed that genes with mutations associated with higher expression of either or both the two MRs are involved in or be annotated with DNA-directed DNA polymerase (*POLD1*, *POLE*, *POLQ*), chromatin regulator (*ATRX*, *KDM6A*, *KMT2C*, *KMT2B*, *RB1*), DNA repair (*ATRX*, *MSH2*, *TP53BP1*, *POLE*, *POLQ*), DNA damage (*ATRX*, *MSH2*, *TP53BP1*, *POLE*, *POLQ*), pathways in cancer (*EGFR*, *EP300*, *MSH2*, *TP53*, *RB1*, *CTNNA1*), viral carcinogenesis (*EP300*, *DDX3X*, *TP53*, *RB1*), tumor suppressor (*MSH2*, *TP53*, *RB1*) and cell cycle (*EP300*, *TP53*, *RB1*) (Figs. 1 g-h, Fig. 6d and a full list in supplementary Table 8).

### Expression of *TOP2A* and *CENPF* in CC compared to other cancer types

**Fig. 7:**
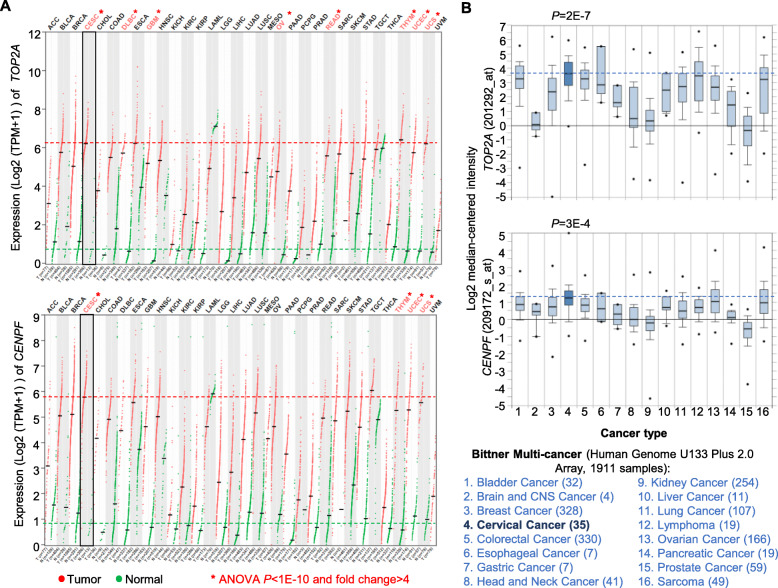
Expression of *CENPF* and *TOP2A* in CC compared to other cancer types. **a** Expression of *TOP2A* and *CENPF* in 33 cancer types (red) and matched normal tissues (green). Data were from TCGA and GTEx analyzed using GEPIA2. Comparisons with ANOVA *P* < 1E-10 and fold change> 4 were highlighted in red text and marked with a *. Red and green horizontal dotted lines reflect the median value of expression for CESC and normal cervical tissue respectively. Abbreviations for cancer types are: ACC, Adrenocortical carcinoma; BLCA, Bladder Urothelial Carcinoma; BRCA, Breast invasive carcinoma; CESC, Cervical squamous cell carcinoma and endocervical adenocarcinoma; CHOL, Cholangio carcinoma; COAD, Colon adenocarcinoma; DLBC, Lymphoid Neoplasm Diffuse Large B-cell Lymphoma; ESCA, Esophageal carcinoma; GBM, Glioblastoma multiforme; HNSC, Head and Neck squamous cell carcinoma; KICH, Kidney Chromophobe; KIRC, Kidney renal clear cell carcinoma; KIRP, Kidney renal papillary cell carcinoma; LAML, Acute Myeloid Leukemia; LGG, Brain Lower Grade Glioma; LIHC, Liver hepatocellular carcinoma; LUAD, Lung adenocarcinoma; LUSC, Lung squamous cell carcinoma; MESO, Mesothelioma; OV, Ovarian serous cystadenocarcinoma; PAAD, Pancreatic adenocarcinoma; PCPG, Pheochromocytoma and Paraganglioma; PRAD, Prostate adenocarcinoma; READ, Rectum adenocarcinoma; SARC, Sarcoma; SKCM, Skin Cutaneous Melanoma; STAD, Stomach adenocarcinoma; TGCT, Testicular Germ Cell Tumors; THCA, Thyroid carcinoma; THYM, Thymoma; UCEC, Uterine Corpus Endometrial Carcinoma; UCS, Uterine Carcinosarcoma; UVM, Uveal Melanoma; **b** Expression analysis of *TOP2A* and *CENPF* using Bittner Multi-cancer Dataset analyzed by Oncomine. The expression in cervical cancer (CC, group 4) was compared to all other cancer types using Student’s *t*-test. *P*-values are indicated. The blue horizontal dotted lines reflect the median value of the expression in CC

Finally, we asked the question whether the two MRs (*TOP2A* and *CENPF*) are especially significant for CC compared to other cancer types. Using GEPIA2, a web server integrating TCGA and the GTEx data for 33 cancer types, we found that *TOP2A* and *CENPF* expression are higher in tumor than normal tissues in most of cancer types except for LAML, which showed opposite trend for both MRs. However, cervical squamous cell carcinoma and endocervical adenocarcinoma (CESC) is one of the several cancer types (8 and 4 for *TOP2A* and *CENPF*, respectively) which showed most significant expression increase (ANOVA *P* < 1E-10 and fold change> 4) in tumor compared to normal tissues (Fig. 7a). Only three other cancer types (THYM, UCEC and UCS) showed similar magnitude of expression change for both MRs. By analyzing “Bittner Multi-cancer”, a multi-cancer microarray dataset using Oncomine, we observed that *TOP2A* and *CENPF* expression was higher (Student’s *t*-test, *P* = 2E-7 and 3E-4 respectively) in CC than other 15 cancer types (Fig. 7b), confirming the RNA-seq data from TCGA and the GTEx.

## Discussion

In current study, *TOP2A*-*CENPF* was identified as a synergistic MR pair in CC. Both MRs showed increased expression and activity in CC. *TOP2A* encodes DNA topoisomerase II alpha, an enzyme that controls and alters the topologic states of DNA, involves in processes such as chromosome condensation, chromatid separation, and the relief of torsional stress during DNA transcription and replication. *TOP2A* was frequently identified as a top overexpressed gene in CC [26, 27, 31–33]. Unsurprisingly, *TOP2A* is considered a late marker in CC and immunohistochemistry or RT-PCR analysis of *TOP2A* combined with other markers (e.g. *MCM2*, *Ki*-*67*, *CDKN2A*) could be used in CC screening which increases the sensitivity or specificity of the diagnosis compared to the high risk HPV test alone [34–37]. The over-expression of *TOP2A* seems to correlate with the grade of cervical intraepithelial neoplasia (CIN), but does not predict prognosis in CC [38, 39]. Consistent with these, in this study, we didn’t observe any significant association between *TOP2A* expression and HPV status, HPV subtype, metastasis or survival among CC samples. Only LVSI and LN positive is associated with higher *TOP2A* expression (Fig. 5c), indicating a potential link with invasion. *CENPF* encodes centromere protein F, that associates with the centromere-kinetochore complex. CENPF is a cell cycle associated nuclear antigen with maximal expression in G2/M-cells. To date, there is no reported functional role of CENPF implicated in CC. However, *CENPF* was reported to be overexpressed, drive tumorigenesis and/or associated with poor prognosis in several human malignancies, including breast cancer [40], prostate cancer [41–44], bladder cancer [45], gastric cancer [46], hepatocellular carcinoma [47–49], pancreatic carcinoma [50]. In this study, higher *CENPF* expression was found to be significantly associated with metastasis compared to CC tissues (Fig. 5a, b). Higher *CENPF* expression is also associated with shorter survival although the *P*-value didn’t reach significance level (supplementary Figure 3). All these suggested that *CENPF* might also be related to tumor progression to metastasis stage and predict poor prognosis in CC. Interestingly, when considering the average expression of *TOP2A* and *CENPF*, both metastasis and LVSI and LN positive are significantly associated with higher average expression (Fig. 5a-c), indicating that a combined feature of the two MRs might be a better marker for CC prognosis.

According to the information from The Human Protein Atlas (http://www.proteinatlas.org) [26], both TOP2A and CENPF proteins have medium to high level of expression in most of CC tissue samples based on the immunohistochemistry results (supplementary Figure 4 and 5). These results confirmed our analysis based on RNA expression and predicted protein activity. Both of them are localized in nucleoplasm (supplementary Figure 6A-B). Both of them show cell cycle dependent expression and are expressed commonly during S and/or G2 phases (supplementary Figure 6C-F). These data at least make it possible that these two proteins are acting synergistically in CC cells. The localization and the cell cycle dependency of their expression are also consistent with the functions of their common regulons: e.g., cell cycle, DNA replication/repair, kinesin complex, chromosome etc. (Fig. 4c). A protein-protein association network analysis of TOP2A, CENPF and their common regulons also suggested a close association of these proteins (supplementary Figure 7). Although a physical interaction between TOP2A and CENPF has not been experimentally determined, many other proteins have shown such interaction. It is also possible that TOP2A and CENPF interact indirectly with each other through other proteins (supplementary Figure 7).

*TOP2A* and *CENPF* are altered (somatic mutation or copy number alternations) in only a small fraction (1 and 5%) of CC patients (supplementary Figure 8). DNA methylation does not seem to correlate with their expression level based on analysis of ~ 800 cancer cell lines (supplementary Figure 9, 10). However, their high expression level is associated with CC patients with missense mutations of 28 genes (Fig. 6a, c, supplementary Table 7). Totally 67 (35%) of the 191 samples have missense mutations in at least one of these 28 genes. And 131 (67%) of the 191 samples have somatic mutations or copy number alternations in the 28 genes or *CENPF* or *TOP2A* (supplementary Figure 8). Many of the 28 genes are directly involved in DNA metabolism (DNA damage, DNA repair, DNA polymerase) or cell cycle. Some are involved in viral carcinogenesis, tumor suppressor and cancer pathways etc., which may eventually drive cancer progression. These data suggest that the two master regulators (*TOP2A* and *CENPF*) may “collect” the somatic mutation effect of many other genes in different cancer-related pathways and with increased expression, promote cell proliferation and tumor progression. Persistent infections of HPV were thought to contribute to 95% of CC cases [51]. One mechanism of HPV to cause CC is that the viral oncogenes inactivate tumor suppressors p53 and RB, leading to increased genomic instability and accumulation of somatic mutations [51]. In this study, we found that missense mutations of *TP53* and *RB1* but not HPV status are associated with higher expression of *TOP2A* and/or *CENPF* (Fig. 6b, supplementary Figure 1). These data may suggest that the high expression of *TOP2A* and/or *CENPF* are hallmarks of CC regardless of HPV status. HPV negative CC may develop tumor through other mechanisms (e.g. accumulate mutations to inactivate *TP53* or *RB1*). Thus, detecting *TOP2A* and/or *CENPF* expression in clinical may decrease the false negative rate of CC diagnosis by HPV test alone.

We found that the two synergistic MRs *TOP2A* and *CENPF* have both the high magnitude of increase of expression (vs. normal tissue) and the high absolute expression level in CC compared to many other cancer types (Fig. 7). This may suggest that these two genes are potential anticancer drug targets in CC. The enzyme encoded by *TOP2A* has been designed as the target for several anticancer agents and a variety of mutations in this gene have been associated with the development of drug resistance [52]. Small molecule compounds targeting CENPF have not been reported, possibly attributed by the difficulty to target a non-enzyme target. However, reports have shown that silencing *CENPF* using in vitro models abolished invasion in gastric cancer [46], resulted in the cell cycle arrest at G2/M checkpoint in hepatocellular carcinoma [47], reduced levels of epithelial-mesenchymal transition markers, inhibited cell proliferation, migration, and invasion, reduced global bio-energetic capacity and altered the global metabolic profiles in prostate cancer [43, 53]. It remained to be validated whether silencing/inhibiting either *CENPF* or both *CENPF* and *TOP2A* will have a strong phenotypic consequence in CC using an in-vitro or/and in vivo model. If these targets can be validated, drugs (an antisense oligo, antibody, etc.) could be developed targeting *CENPF* with or without a combination of a small molecule drug targeting TOP2A in future CC treatment.

To explore more related to drug selection in clinical use for cancer patient, we queried CMap database using CLUE and identified potential perturbations that could reverse the over-expression signature of *TOP2A*, *CENPF* and their common regulons (supplementary Table 9). Unsurprisingly, CDK inhibitor purvalanol-a, aminopurvalanol-a, JAK3-inhibitor-VI, and topoisomerase inhibitor, cell cycle inhibitor etoposide are top listed compounds (CPs) that showed a strong negative connectivity scores in 8 cancer cell lines. The “Cell Cycle Inhibition GOF” is the top perturbation class (PCL), consistent with the function of the two MRs and their common regulons. These data may help the clinicians to select better drugs for cancer patients with an over-expression signature of the two MRs and their common regulons. This also suggests that developing or selecting drugs targeting the signal pathway they involved in might be a promising approach for CC and other cancer treatment.

## Conclusions

In conclusion, our analysis suggested that *TOP2A* and *CENPF* are MRs that are overexpressed and activated in CC and synergistically regulate a common set of regulons, with functions related to cell cycle, DNA replication/repair, kinesin complex etc. Their high expression are linked to metastasis and the mutation status of a set of genes including a few tumor suppressors. They may serve as biomarkers for future CC diagnosis. They are also potential anticancer drug targets for future drug discovery. Our discovery could also provide guidance for clinicians to select anti-cancer drugs for CC patients. Finally, some of the observations were also observed in other cancer types, suggesting a potential wider impact of our study.

## Supplementary information

**Additional file 1: Supplementary Table 1**: MR DA and DE signatures for the 10 datasets. “MR_set”, categories of functions of MRs (TF, transcriptional factor; TFcoFac, transcriptional co-factors; SigPathway, signal pathway); “regulon_size”, number of regulons for a MR; “rank_cat”, category of ranking. For example, 100 indicates that the MR is a common MR of the top 100 activated MRs in the 5 discovery datasets. -100 indicates that the MR is a common MR of the top 100 suppressed MRs in the 5 discovery datasets. 0 indicates the MR is not the common top MRs in the 5 discovery datasets. “nes.”, the NES score of a MR in a dataset calculated using VIPER, representing the DA signature. “pval.”, the *P*-value of the NES from the VIPER analysis. “t.”, the value representing the DE signature (e.g. the *t*-statistics for microarray). “DiscoverySet_DA-DE_consistent”, whether the DA and DE are consistent. The DA-DE Consistency set was selected using |DE| value> 3 and same sign as DA in all the five discovery datasets. **Supplementary Table 2**: DE signatures of the common regulons of *TOP2A* and *CENPF* for the 10 datasets. “MOR_sign”, sign of MOR (positive (1) or negative (− 1) MOR for both *TOP2A*-regulons and *CENPF*-regulons). “DE.average”, the average values of the DE values for the 10 datasets. **Supplementary Table 3**: Network of *TOP2A*, *CENPF* and their regulons. “Regulator” is the gene ID of a MR (here *CENPF* or *TOP2A*). “interaction” is always “regulate”. “Target” is the regulon of a regulator. “MOR”, mode of regulation, ranges between − 1 and + 1. “likelihood”, range from 0 to 1, an edge weight that indicates how strong the mutual information for an edge is. All information was extracted from the aracne.networks R package. **Supplementary Table 4**: “Functional Annotation Clustering” analysis of the 34 *TOP2A-CENPF* common regulons on DAVID 6.8 website. See DAVID website for details of methods used and interpretation of header names. “Category”, the gene annotation category. UP_KEYWORDS, UniProt Keywords annotation of genes; UP_SEQ_FEATURE, UniProt sequence feature annotation of genes; GOTERM_BP_DIRECT, Gene ontology biological process annotation; GOTERM_CC_DIRECT, Gene ontology cellular component annotation. **Supplementary Table 5**: *CENPF* and *TOP2A* expression in dataset Biewenga_2008 (GSE7410). The expression data were extracted from GEO database using GEO2R tool. The probe ID is indicated after the gene symbol. “Avg_Gex”, the average expression value of *CENPF* and *TOP2A*. “sample_type”, sample type (N, healthy cervical tissue; C, Early stage cervical tumour without lymph node metastasis; M, Early stage cervical tumour with lymph node metastasis). **Supplementary Table 6**: *CENPF*, *TOP2A* expression and clinical, molecular data in CC samples in TCGA. The clinical and molecular feature information were from the supplementary Table 1 and 2 of the publication: The Cancer Genome Atlas Research Network et al., 2017. The expression values of *CENPF* and *TOP2A* were downloaded from TSVdb. **Supplementary Table 7**: Somatic mutations associated with expression of *CENPF* and *TOP2A* in CC TCGA data. Each row is an association of a gene (Gene.mutation) with missense or LOF mutations and expression of *CENPF* or *TOP2A* tested using Wilcoxon Rank-Sum test. “delta. Median.Exp” is the difference of average expression of samples with mutation and samples without mutation. “Direction.Exp” indicates whether the samples with mutation has higher or lower expression than the samples without mutation. *P*-value is indicated in “Wilcox.P” column. Only associations with a *P* < 0.1 were included in the list. **Supplementary Table 8**: DAVID “Functional Annotation Clustering” analysis of the genes with mutations associated with high or low expression of *TOP2A* or *CENPF*. See DAVID website for details of methods used and interpretation of header names. “Category”, the gene annotation category. UP_KEYWORDS, UniProt Keywords annotation of genes; KEGG_PATHWAY, KEGG pathway annotation of genes. **Supplementary Table 9**: Connections of expression signature of *TOP2A*, *CENPF* and their common regulons with all expression signatures in Connectivity Map (CMap) using the QUERY app of CLUE. Each row is a perturbation in CMap. Id, perturbation ID; type, type of perturbation (CP, compound; KD, gene knockdown; OE, gene over-expression; PCL, perturbation class); HA1E, PC3, VCAP, A375, A549, HCC515, HT29, MCF7, HEPG2, the connectivity score for the indicated cell lines; summary, summary connectivity score of all cell lines; Average_CancerCells, average connectivity score of 8 cancer cell lines (except for HA1E (human kidney epithelial cell), all other cell lines are cancer cell lines). Name, perturbation name; Description, perturbation description; Target, the target of a compound; “belongs_to”, the PCL the perturbation belongs to. The table was sorted based on ascending Average_CancerCells. The top rows represent perturbations thar reverse (downregulate) the expression of *Top2A*, *CENPF* and their common regulons. The negative connectivity scores from − 95 to − 100 were formatted in Excel in blue background with increasing darkness.

**Additional file 2: Supplementary Figure 1**. Association of *CENPF* and *TOP2A* expression with category clinical features (TCGA data). The clinical category with > 5 samples were used for comparison. The groups were sorted by median expression from low to high. The number of samples in each group is indicated above the plot (using “# = xx xx” format, in which “xx” represent sample number matching the sorted groups). The groups with the lowest and highest median expression were compared using Wilcoxon Rank-Sum test. The *P*-values are indicated on the top of the plot. *P* < 0.05 was indicated by a *. **Supplementary Figure 2**. Correlation of *CENPF* and *TOP2A* expression (y axis) with continuous clinical features (x axis) (TCGA data). A linear regression line was draw. The Spearman correlation coefficient (r) and *P*-value are indicated above each plot. *P* < 0.05 was marked with an arrow. **Supplementary Figure 3**. Survival analysis of *CENPF* and *TOP2A* expression. The TCGA samples were separated to two groups (exp-high in red and exp-low in blue) using the 80th percentile of expression of *CENPF* or *TOP2A*. *P*-value is based on the log-rank test. Confidence intervals were shown as shaded areas. **Supplementary Figure 4**. Protein expression of TOP2A in CC tissues as detected by immunohistochemistry (data retrieved from The Human Protein Atlas). (A) Summary of three antibody staining results for 11 patients as detected by antibody HPA006458 or 12 patients as detected by antibodies HPA026773 and CAB002448 (full bar represents all patients). (B) An example of staining image from a patient with cervix squamous cell carcinoma. (C) An example of staining image from a patient with cervix adenocarcinoma. **Supplementary Figure 5**. Protein expression of CENPF in CC tissues as detected by immunohistochemistry (data retrieved from The Human Protein Atlas). (A) Summary of two antibody staining results for 11 patients per antibody (full bar represents all patients). (B) An example of staining image from a patient with cervix squamous cell carcinoma. (C) An example of staining image from a patient with cervix adenocarcinoma. **Supplementary Figure 6**. Protein expression in subcellular localization and cell cycle for CENPF and TOP2A (data retrieved from The Human Protein Atlas). (A) Staining of Hela cell shows that CENPF is mainly localized in nucleoplasm. (B) Staining of U2 OS cell shows that TOP2A is mainly localized in nucleoplasm. (C-D) Staining of U-2 OS FUCCI cells, to characterize the cell cycle dependency of the protein expression pattern. CENPF is mostly expressed during S and/or G2. (E-F) Similar to C-D unless that protein TOP2A is shown to be expressed during S and/or G2 and during Mitosis. **Supplementary Figure 7**. Protein-Protein association network of TOP2A, CENPF and their common regulons. **Supplementary Figure 8**. OncoPrint visualization of somatic mutations, copy number alterations for the 28 genes (rows) with missense mutation associated with higher expression of *CENPF* and/or *TOP2A* in 191 cervical squamous cell carcinoma and endocervical adenocarcinoma (TCGA, Firehose Legacy) samples (columns). The mutation and gene expression profile of *CENPF* and *TOP2A* are also shown. 131 (69%) of queried patients/samples have at least one queried gene altered. **Supplementary Figure 9**. Correlation of gene expression and promoter region methylation of TOP2A in 789 cancer cell lines. (A) Scatter plot, each cancer type is shown in a different color. A linear regression line is drawn for each cancer type. (B) Table of correlation coefficients and linear regression *P*-values for different cancer types. **Supplementary Figure 10**. Correlation of gene expression and promoter region methylation of CENPF in 823 cancer cell lines. (A) and (B) Similar to supplementary Figure 9 except that CENPF is shown.

## Data Availability

Overall survival datasets **Table:** 

Data Name	Data type	Database	ID or URL
Biewenga_2008	**Microarray**	**GEO**	**GSE7410**
Boon_2015	**Microarray**	**GEO**	**GSE63514**
Guardado_2012	**Microarray**	**GEO**	**GSE29570**
Medina_2014	**Microarray**	**GEO**	**GSE52903**
Zhai_2007	**Microarray**	**GEO**	**GSE7803**
Li_2018	**RNA-seq**	**GEO**	**GSE107472**
Pappa_2015	**Microarray**	**GEO**	**GSE63678**
Pyeon_2007	**Microarray**	**GEO**	**GSE6791**
Scotto_2008	**Microarray**	**GEO**	**GSE9750**
Sun_2014	**Microarray**	**GEO**	**GSE55940**
human reference genome	**DNA sequences**	**UCSC genome browser**	**http://hgdownload.soe.ucsc.edu/goldenPath/hg19/chromosomes/**
aracne.networks	**R package**	**Bioconductor**	**https://bioconductor.org/packages/release/data/experiment/html/aracne.networks.html**
viper	**R package**	**Bioconductor**	**https://www.bioconductor.org/packages/release/bioc/html/viper.html**
TOP2A or CENPF gene expression data for TCGA samples	**RSEM (RNA-Seq by Expectation Maximization) values**	**TSVdb**	**http://www.tsvdb.com/index.html. (Click START, query cancer type and gene symbol, click "download data" to download raw data****.)**
clinical and molecular features of CC samples from TCGA	**supplementary table**	**Cancer Genome Atlas Research Network et al., 2017**	**https://static-content.springer.com/esm/art%3A10.1038%2Fnature21386/MediaObjects/41586_2017_BFnature21386_MOESM213_ESM.zip**

## Abbreviations

ANOVA: Analysis of variance
CC: Cervical cancer
CDS: Coding sequence
*CENPF*: Centromere protein F
CIN: Cervical intraepithelial neoplasia
CMap: Connectivity Map
DA: Differential activity
DAVID: The Database for Annotation, Visualization and Integrated Discovery
DE: Differential expression
ECA: Expression contribution to activity
FUCCI: Fluorescence Ubiquitination Cell Cycle Indicator
GEO: Gene Expression Omnibus
GEPIA2: Gene Expression Profiling Interactive Analysis 2
GO: Gene ontology
GTEx: The Genotype-Tissue Expression
HPV: Human papillomavirus
KEGG: Kyoto Encyclopedia of Genes and Genomes
LN: Lymph nodes
LOF: Loss-of-function
LVSI: Lymphovascular space invasion
MAPQ: Mapping quality score
MOR: Mode of regulation
MR: Master regulator
N: Normal tissue
NES: Normalized enrichment score
OS: Overall survival
RSEM: RNA-Seq by Expectation Maximization
SMG: Gene with somatic mutations
SRA: Sequence Read Archive
TCGA: The Cancer Genome Atlas
*TOP2A*: DNA topoisomerase II alpha
TPM: Transcript Per Million
VIPER: Virtual Inference of Protein-activity by Enriched Regulon analysis
