# Supplementary material for: TOP2A and CENPF are synergistic master regulators activated in cervical cancer
Source: BMC Med Genomics. 2020 Oct 6;13:145. doi: 10.1186/s12920-020-00800-2 (PMC7541258; doi:10.1186/s12920-020-00800-2)
Supplement: Supplementary file 2 — Additional file 2: Supplementary Figure 1. Association of CENPF and TOP2A expression with category clinical features (TCGA data). The clinical category with > 5 samples were used for comparison. The groups were sorted by median expression from low to high. The number of samples in each group is indicated above the plot (using “# = xx xx” format, in which “xx” represent sample number matching the sorted groups). The groups with the lowest and highest median expression were compared using Wilcoxon Rank-Sum test. The P-values are indicated on the top of the plot. P < 0.05 was indicated by a *. Supplementary Figure 2. Correlation of CENPF and TOP2A expression (y axis) with continuous clinical features (x axis) (TCGA data). A linear regression line was draw. The Spearman correlation coefficient (r) and P-value are indicated above each plot. P < 0.05 was marked with an arrow. Supplementary Figure 3. Survival analysis of CENPF and TOP2A expression. The TCGA samples were separated to two groups (exp-high in red and exp-low in blue) using the 80th percentile of expression of CENPF or TOP2A. P-value is based on the log-rank test. Confidence intervals were shown as shaded areas. Supplementary Figure 4. Protein expression of TOP2A in CC tissues as detected by immunohistochemistry (data retrieved from The Human Protein Atlas). (A) Summary of three antibody staining results for 11 patients as detected by antibody HPA006458 or 12 patients as detected by antibodies HPA026773 and CAB002448 (full bar represents all patients). (B) An example of staining image from a patient with cervix squamous cell carcinoma. (C) An example of staining image from a patient with cervix adenocarcinoma. Supplementary Figure 5. Protein expression of CENPF in CC tissues as detected by immunohistochemistry (data retrieved from The Human Protein Atlas). (A) Summary of two antibody staining results for 11 patients per antibody (full bar represents all patients). (B) An example of staining image from a patien [file 12920_2020_800_MOESM2_ESM.pptx]

## Slide 1
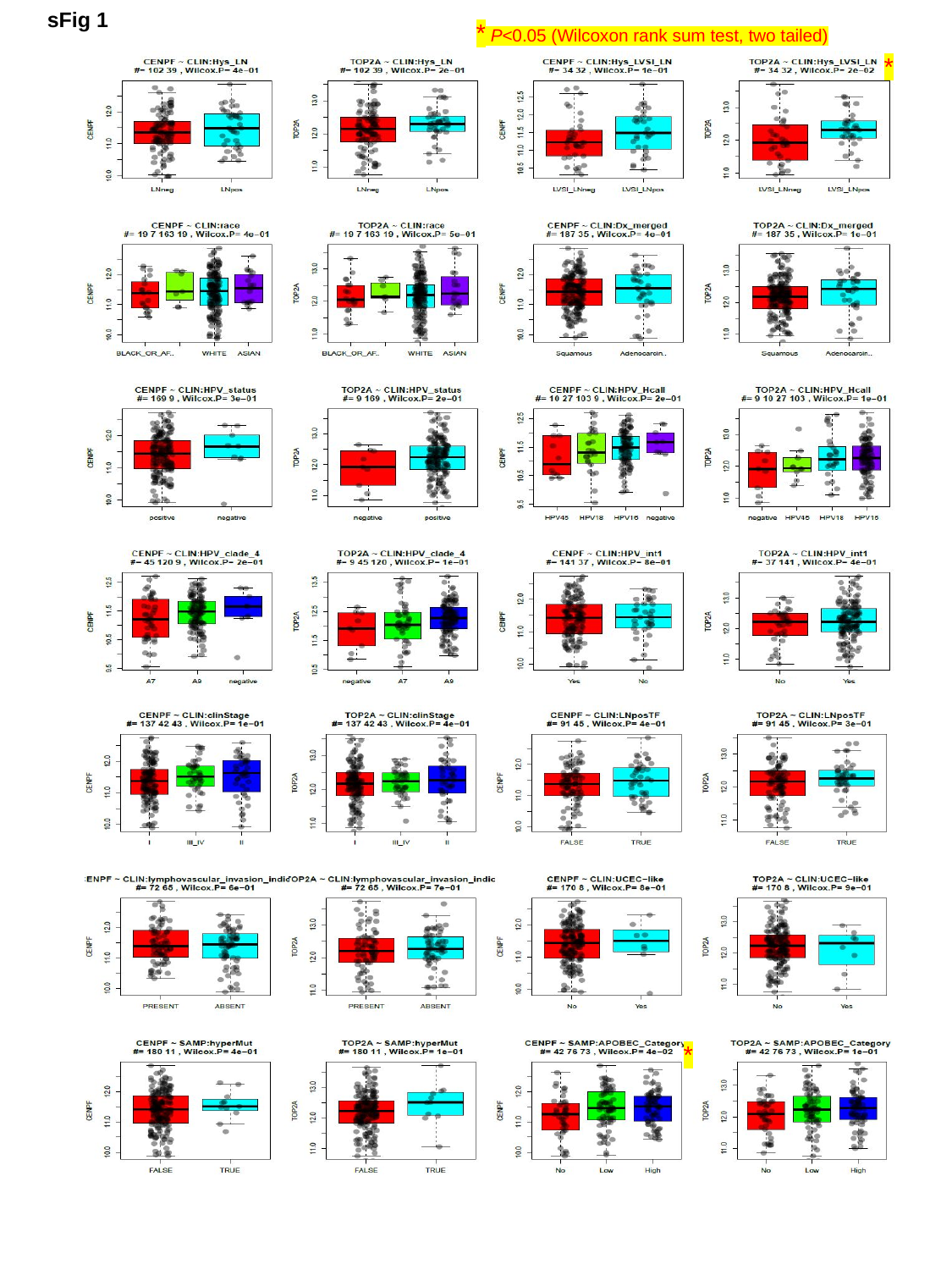

sFig 1
* P<0.05 (Wilcoxon rank sum test, two tailed)
*
*

## Slide 2
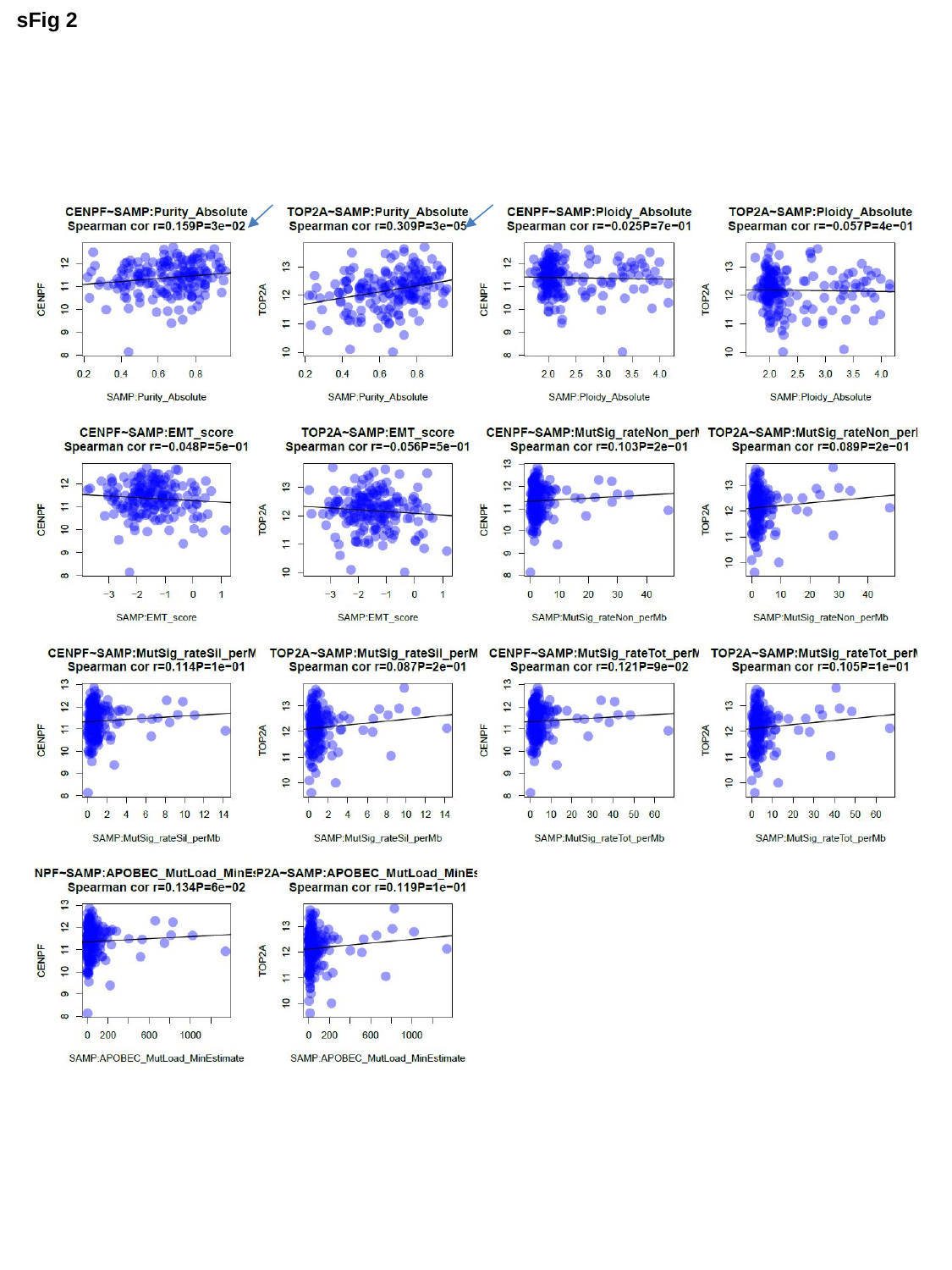

sFig 2

## Slide 3
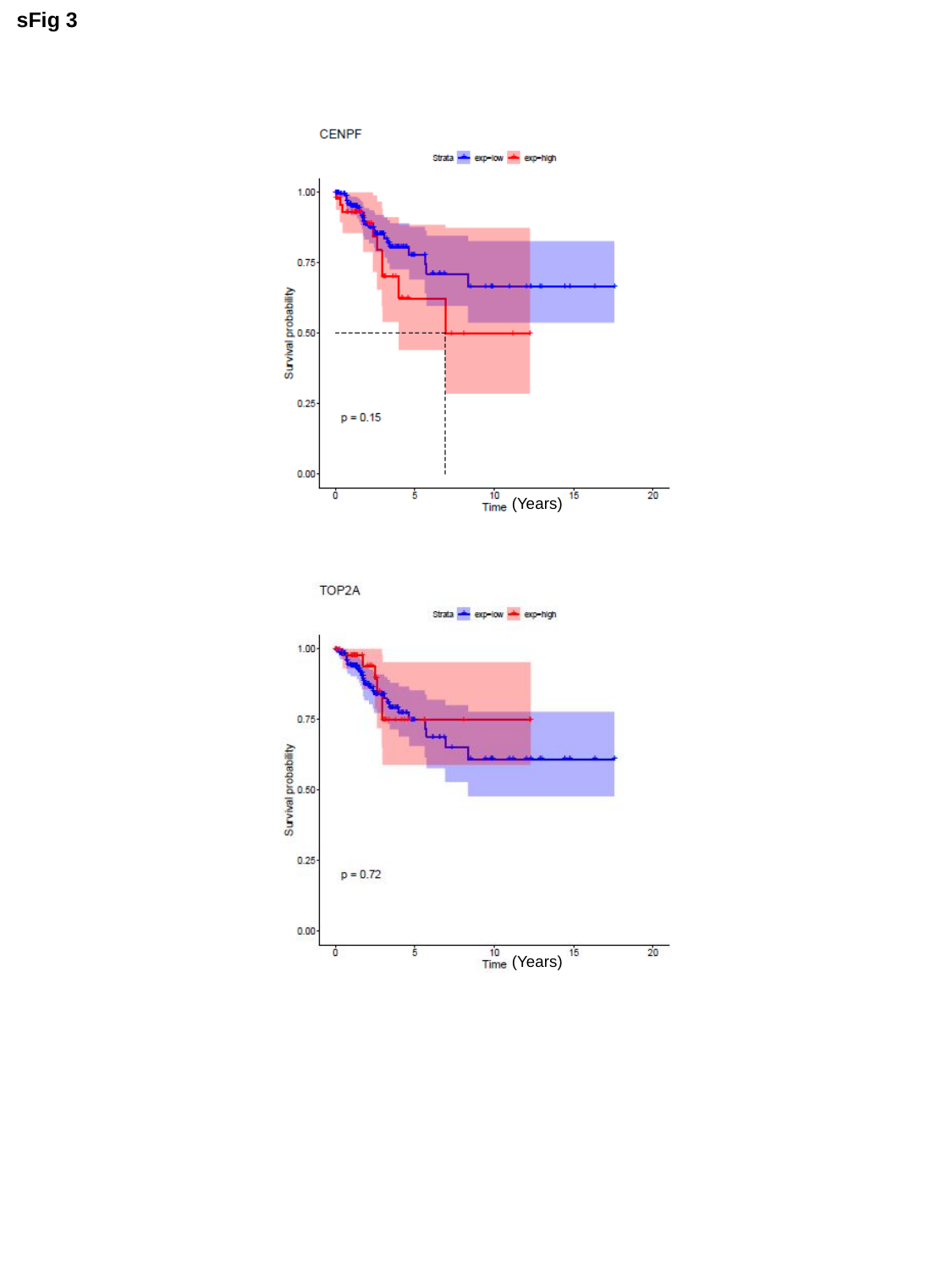

sFig 3
(Years)
(Years)

## Slide 4
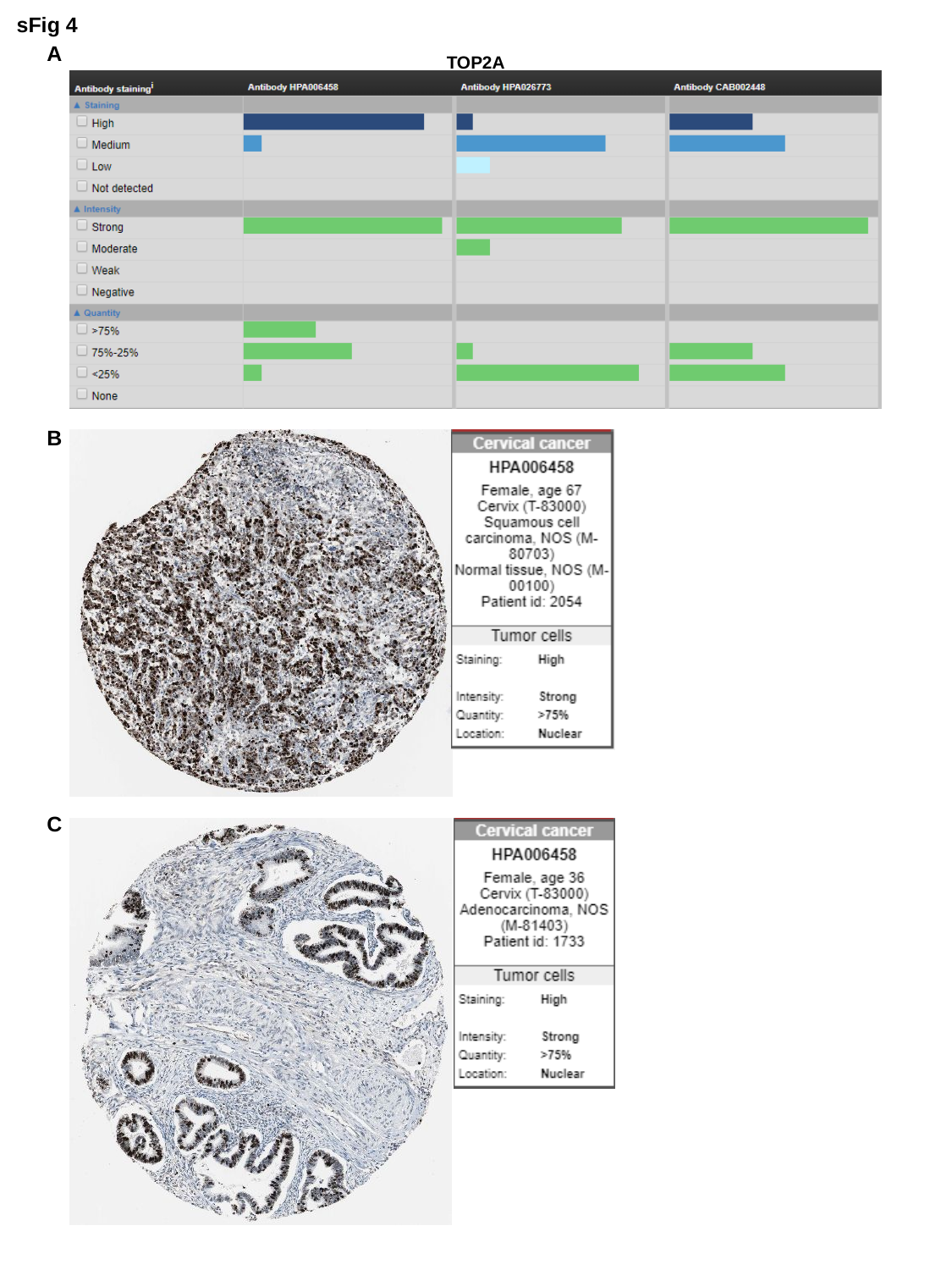

sFig 4
A
TOP2A
B
C

## Slide 5
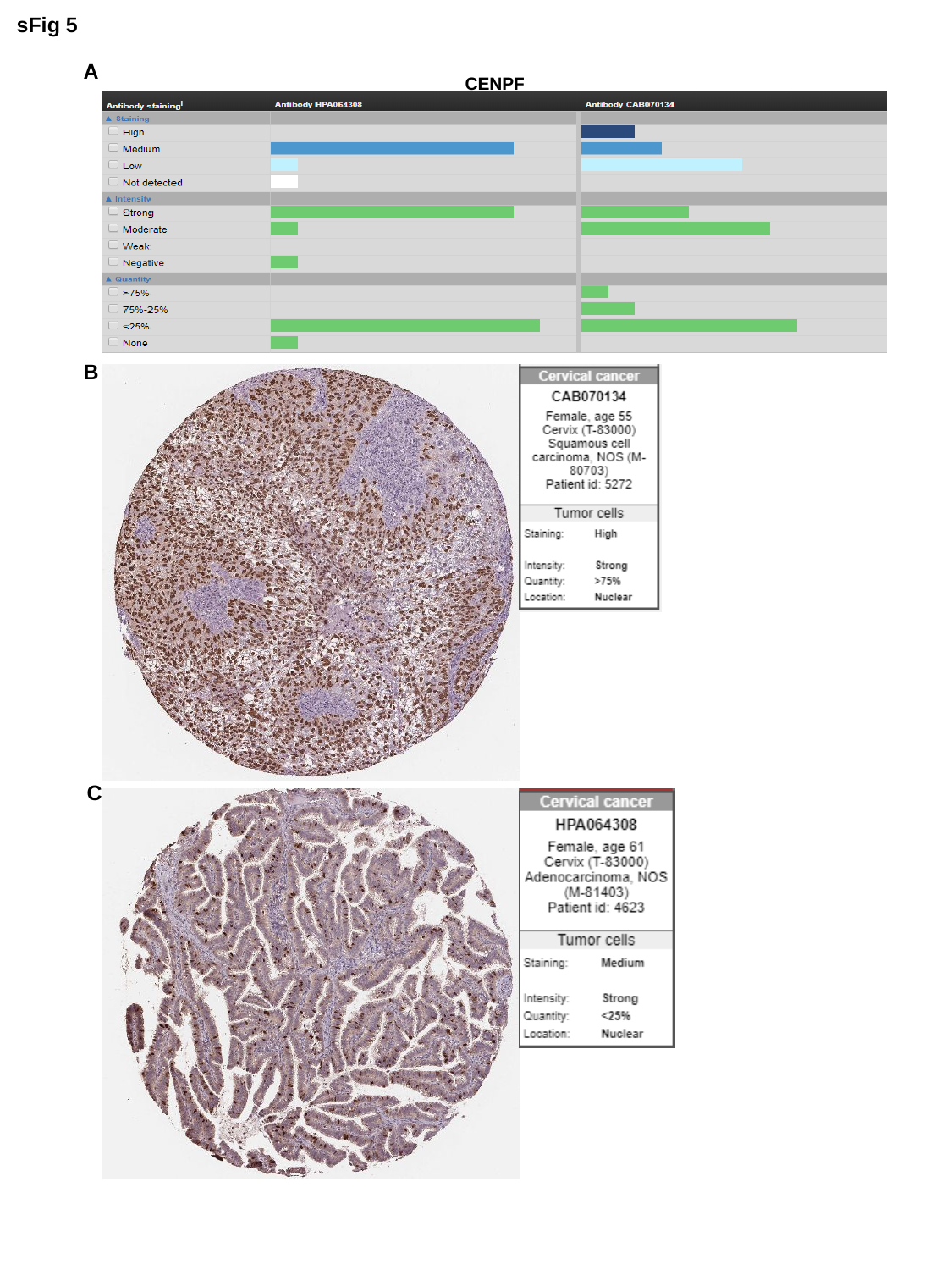

sFig 5
A
CENPF
B
C

## Slide 6
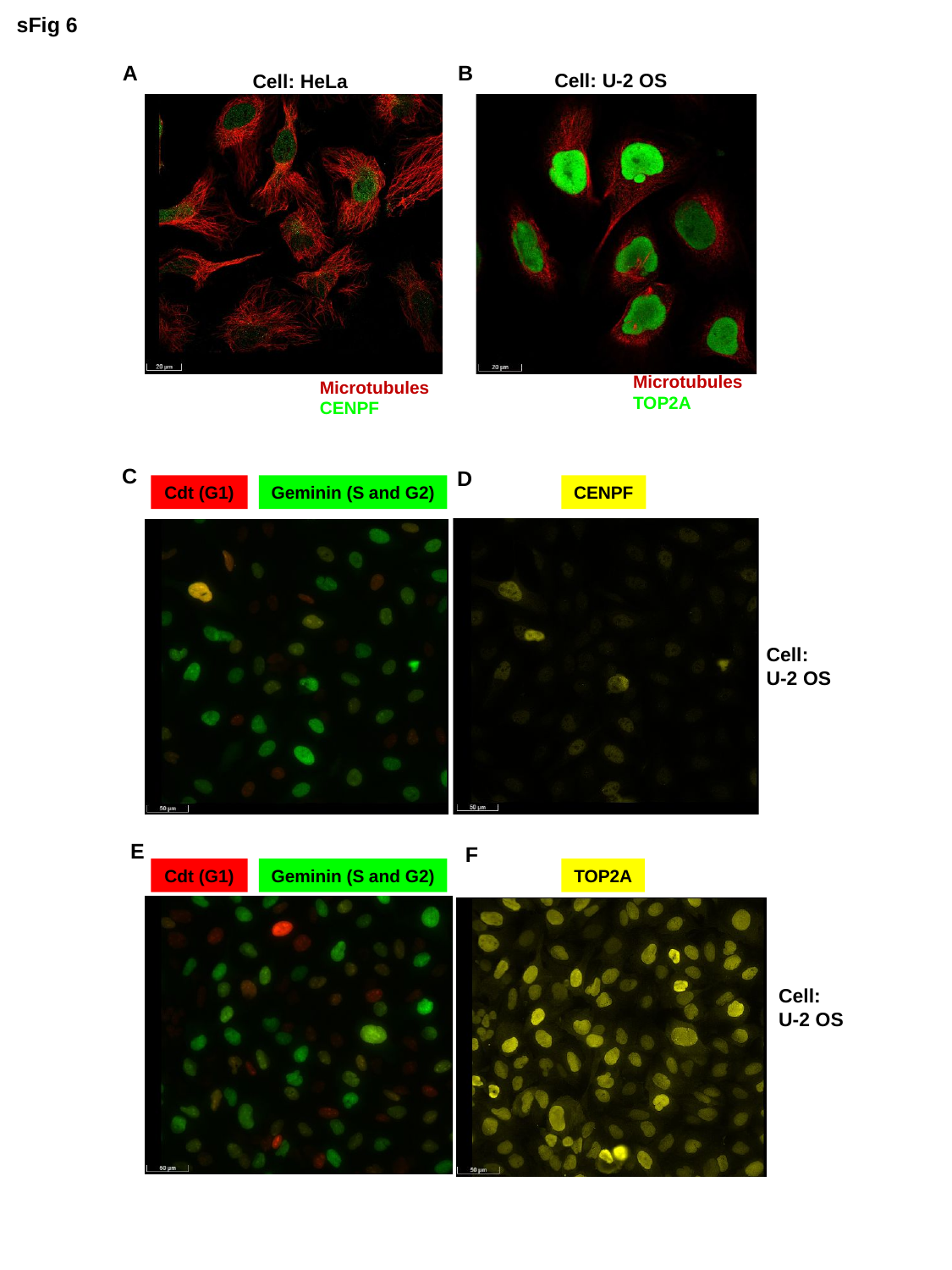

sFig 6
A
B
Cell: U-2 OS
Cell: HeLa
Microtubules
TOP2A
Microtubules
CENPF
C
D
CENPF
Cdt (G1)
Geminin (S and G2)
Cell:
U-2 OS
E
F
TOP2A
Cdt (G1)
Geminin (S and G2)
Cell:
U-2 OS

## Slide 7
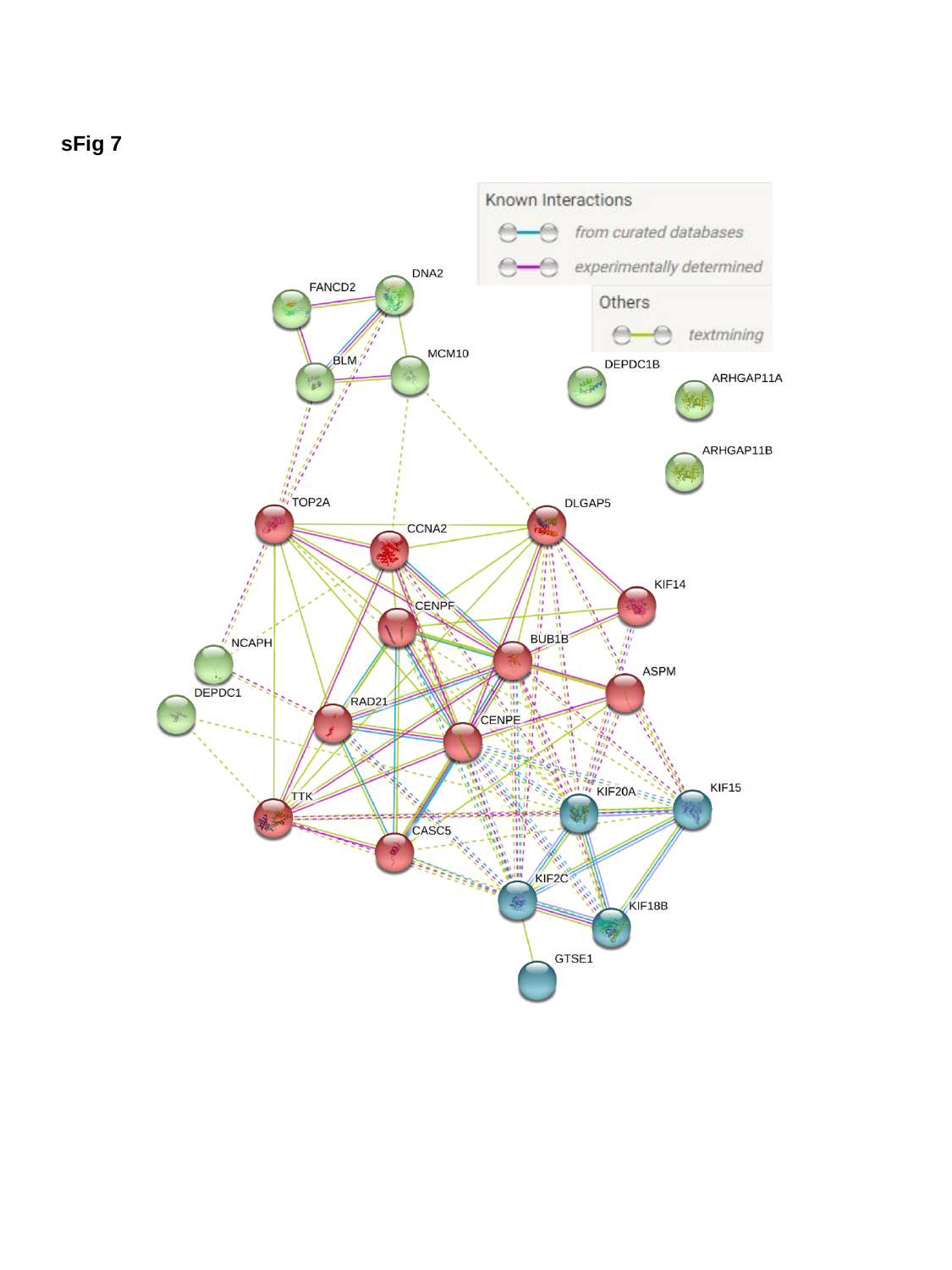

sFig 7

## Slide 8
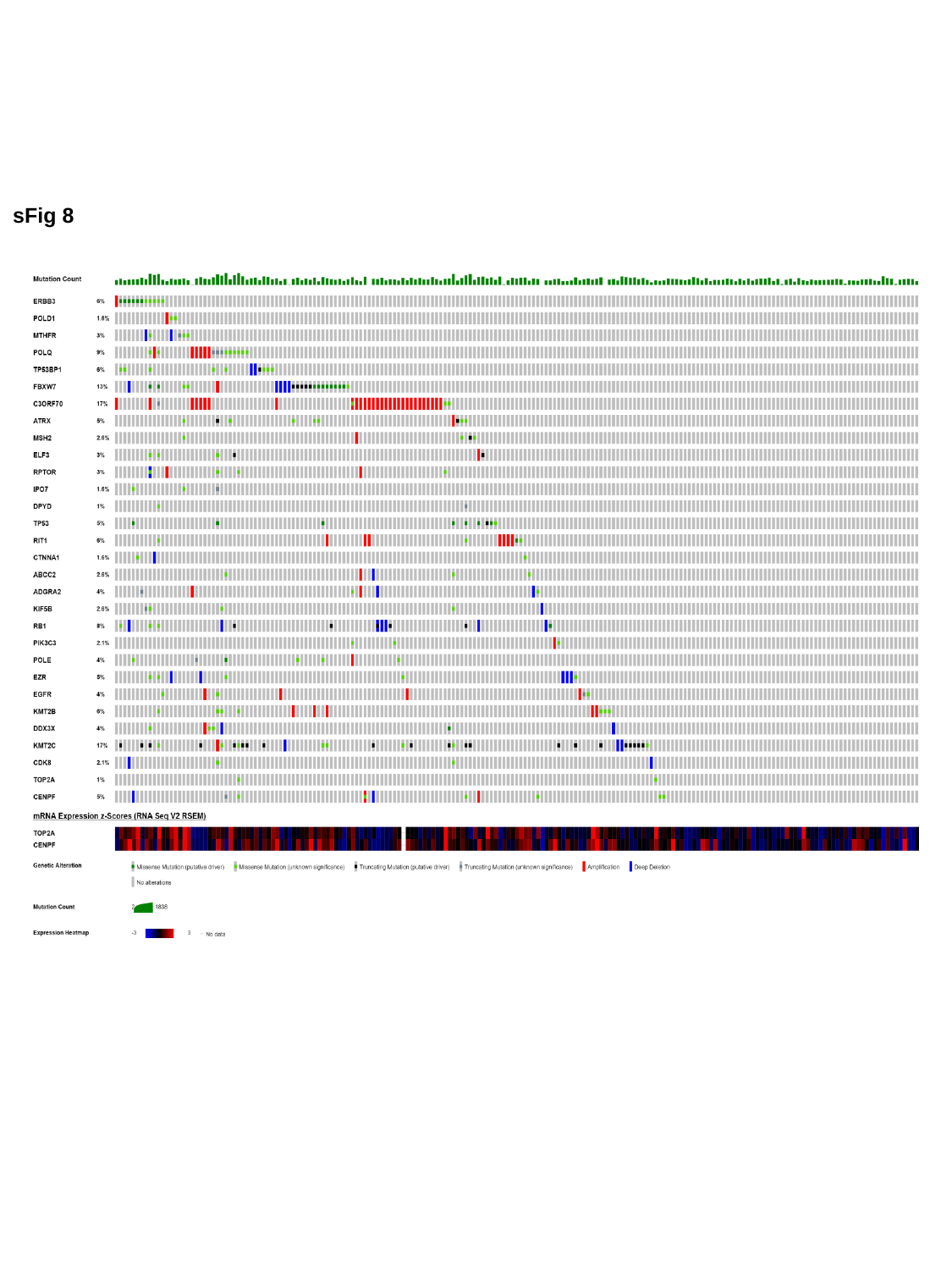

sFig 8

## Slide 9
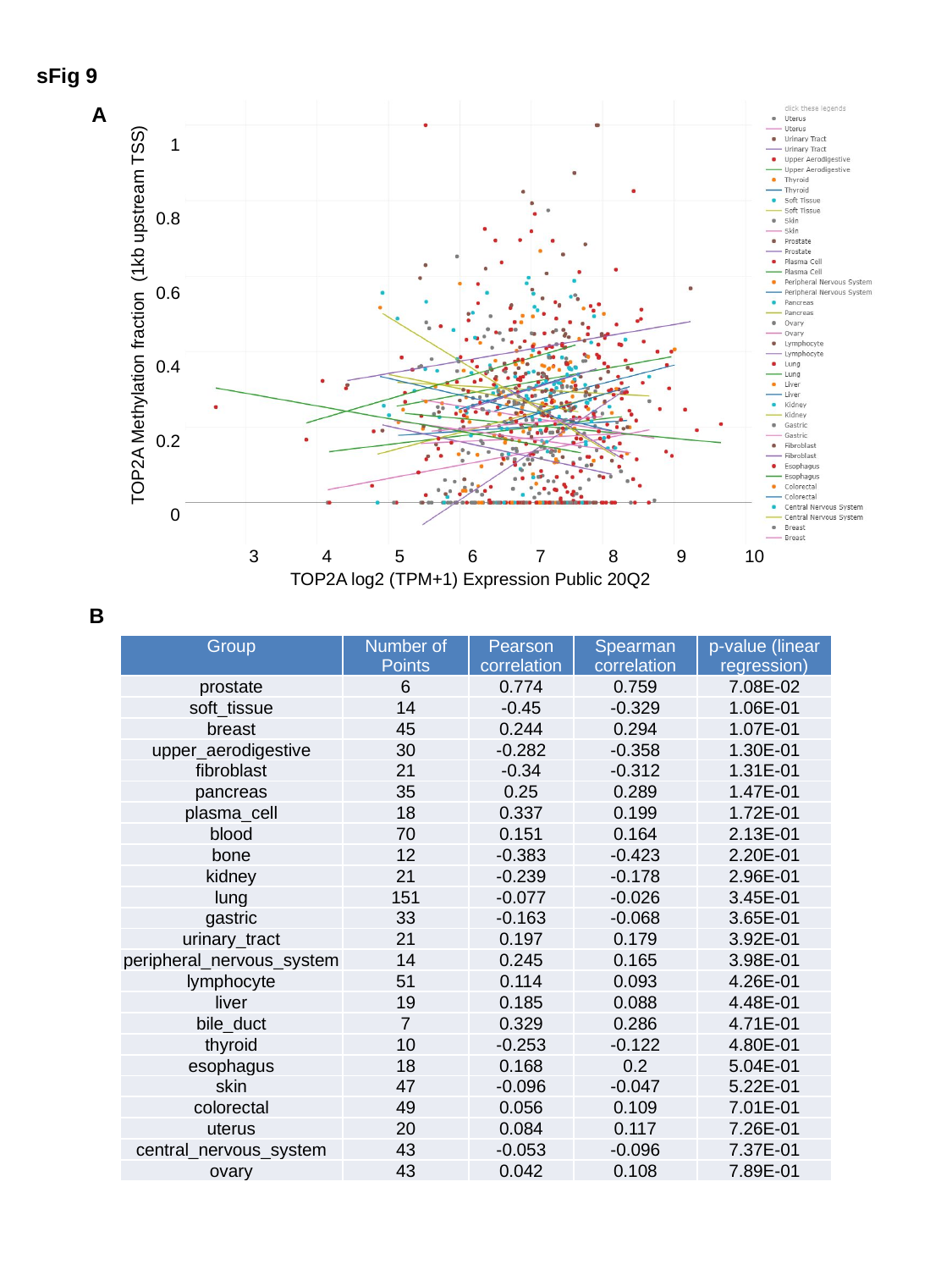

sFig 9
1
0.8
0.6
0.4
0.2
0
A
TOP2A Methylation fraction (1kb upstream TSS)
3 4 5 6 7 8 9 10
TOP2A log2 (TPM+1) Expression Public 20Q2
B
| Group | Number of Points | Pearson correlation | Spearman correlation | p-value (linear regression) |
| --- | --- | --- | --- | --- |
| prostate | 6 | 0.774 | 0.759 | 7.08E-02 |
| soft\_tissue | 14 | -0.45 | -0.329 | 1.06E-01 |
| breast | 45 | 0.244 | 0.294 | 1.07E-01 |
| upper\_aerodigestive | 30 | -0.282 | -0.358 | 1.30E-01 |
| fibroblast | 21 | -0.34 | -0.312 | 1.31E-01 |
| pancreas | 35 | 0.25 | 0.289 | 1.47E-01 |
| plasma\_cell | 18 | 0.337 | 0.199 | 1.72E-01 |
| blood | 70 | 0.151 | 0.164 | 2.13E-01 |
| bone | 12 | -0.383 | -0.423 | 2.20E-01 |
| kidney | 21 | -0.239 | -0.178 | 2.96E-01 |
| lung | 151 | -0.077 | -0.026 | 3.45E-01 |
| gastric | 33 | -0.163 | -0.068 | 3.65E-01 |
| urinary\_tract | 21 | 0.197 | 0.179 | 3.92E-01 |
| peripheral\_nervous\_system | 14 | 0.245 | 0.165 | 3.98E-01 |
| lymphocyte | 51 | 0.114 | 0.093 | 4.26E-01 |
| liver | 19 | 0.185 | 0.088 | 4.48E-01 |
| bile\_duct | 7 | 0.329 | 0.286 | 4.71E-01 |
| thyroid | 10 | -0.253 | -0.122 | 4.80E-01 |
| esophagus | 18 | 0.168 | 0.2 | 5.04E-01 |
| skin | 47 | -0.096 | -0.047 | 5.22E-01 |
| colorectal | 49 | 0.056 | 0.109 | 7.01E-01 |
| uterus | 20 | 0.084 | 0.117 | 7.26E-01 |
| central\_nervous\_system | 43 | -0.053 | -0.096 | 7.37E-01 |
| ovary | 43 | 0.042 | 0.108 | 7.89E-01 |

## Slide 10
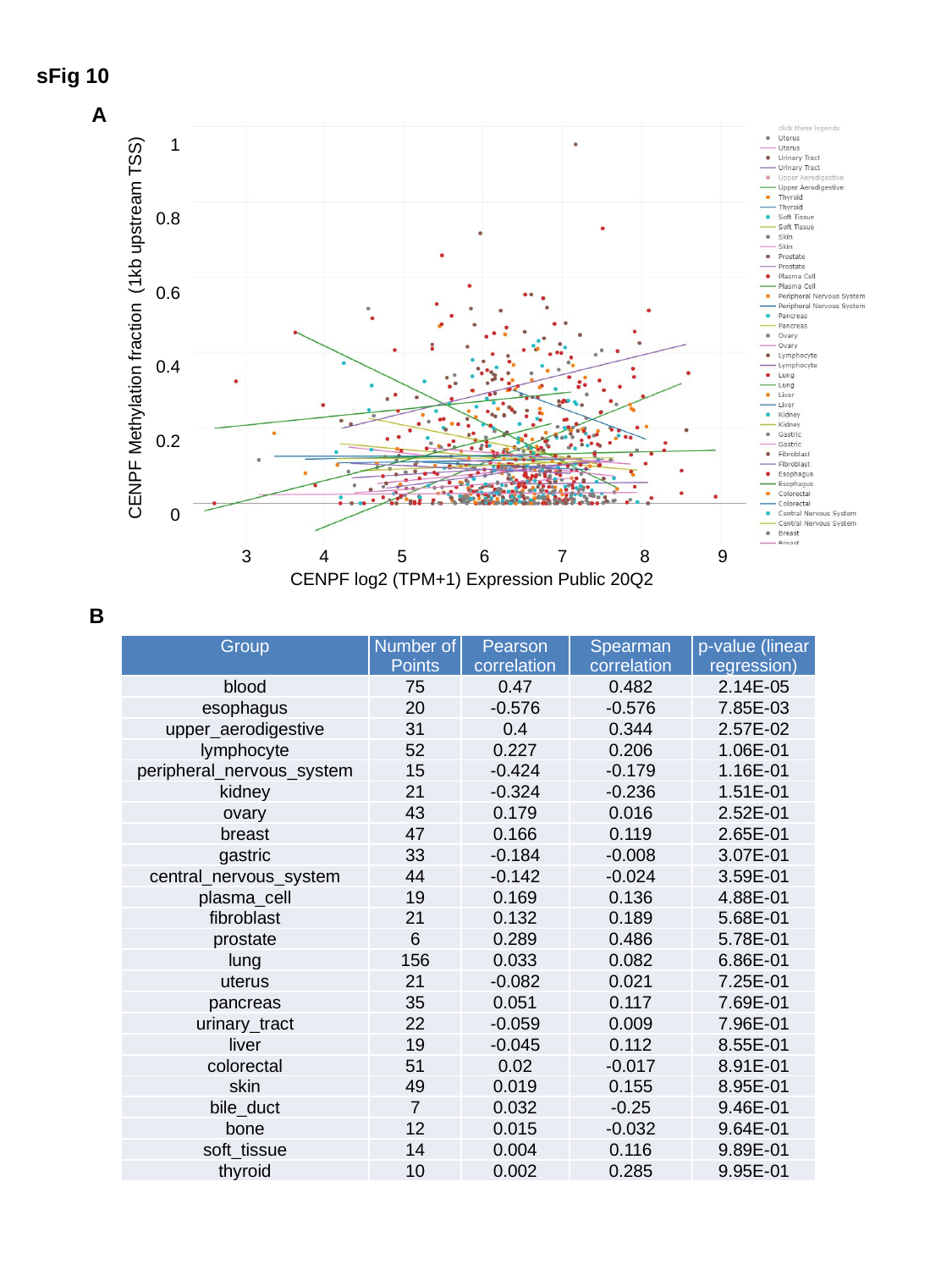

sFig 10
1
0.8
0.6
0.4
0.2
0
A
CENPF Methylation fraction (1kb upstream TSS)
3 4 5 6 7 8 9
CENPF log2 (TPM+1) Expression Public 20Q2
B
| Group | Number of Points | Pearson correlation | Spearman correlation | p-value (linear regression) |
| --- | --- | --- | --- | --- |
| blood | 75 | 0.47 | 0.482 | 2.14E-05 |
| esophagus | 20 | -0.576 | -0.576 | 7.85E-03 |
| upper\_aerodigestive | 31 | 0.4 | 0.344 | 2.57E-02 |
| lymphocyte | 52 | 0.227 | 0.206 | 1.06E-01 |
| peripheral\_nervous\_system | 15 | -0.424 | -0.179 | 1.16E-01 |
| kidney | 21 | -0.324 | -0.236 | 1.51E-01 |
| ovary | 43 | 0.179 | 0.016 | 2.52E-01 |
| breast | 47 | 0.166 | 0.119 | 2.65E-01 |
| gastric | 33 | -0.184 | -0.008 | 3.07E-01 |
| central\_nervous\_system | 44 | -0.142 | -0.024 | 3.59E-01 |
| plasma\_cell | 19 | 0.169 | 0.136 | 4.88E-01 |
| fibroblast | 21 | 0.132 | 0.189 | 5.68E-01 |
| prostate | 6 | 0.289 | 0.486 | 5.78E-01 |
| lung | 156 | 0.033 | 0.082 | 6.86E-01 |
| uterus | 21 | -0.082 | 0.021 | 7.25E-01 |
| pancreas | 35 | 0.051 | 0.117 | 7.69E-01 |
| urinary\_tract | 22 | -0.059 | 0.009 | 7.96E-01 |
| liver | 19 | -0.045 | 0.112 | 8.55E-01 |
| colorectal | 51 | 0.02 | -0.017 | 8.91E-01 |
| skin | 49 | 0.019 | 0.155 | 8.95E-01 |
| bile\_duct | 7 | 0.032 | -0.25 | 9.46E-01 |
| bone | 12 | 0.015 | -0.032 | 9.64E-01 |
| soft\_tissue | 14 | 0.004 | 0.116 | 9.89E-01 |
| thyroid | 10 | 0.002 | 0.285 | 9.95E-01 |
